# Extraction of nickel from NiFe-LDH into Ni_2_P@NiFe hydroxide as a bifunctional electrocatalyst for efficient overall water splitting†
†Electronic supplementary information (ESI) available: Experimental and computational details and additional data. See DOI: 10.1039/c7sc04569g


**DOI:** 10.1039/c7sc04569g

**Published:** 2017-12-21

**Authors:** Fang-Shuai Zhang, Jia-Wei Wang, Jun Luo, Rui-Rui Liu, Zhi-Ming Zhang, Chun-Ting He, Tong-Bu Lu

**Affiliations:** a MOE Key Laboratory of Bioinorganic and Synthetic Chemistry , School of Chemistry , Sun Yat-Sen University , Guangzhou 510275 , China . Email: hechunt@mail2.sysu.edu.cn ; Email: lutongbu@mail.sysu.edu.cn; b Institute of New Energy Materials & Low Carbon Technology , School of Material Science & Engineering , Tianjin University of Technology , Tianjin 300384 , China . Email: zmzhang@email.tjut.edu.cn

## Abstract

The development of highly efficient, low-cost and stable electrocatalysts for overall water splitting is highly desirable for the storage of intermittent solar energy and wind energy sources. Herein, we show for the first time that nickel can be extracted from NiFe-layered double hydroxide (NiFe-LDH) to generate an Ni_2_P@FePO_*x*_ heterostructure. The Ni_2_P@FePO_*x*_ heterostructure was converted to an Ni_2_P@NiFe hydroxide heterostructure (P-NiFe) during water splitting, which displays high electrocatalytic performance for both the hydrogen evolution reaction (HER) and oxygen evolution reaction (OER) in 1.0 M KOH solution, with an overpotential of 75 mV at 10 mA cm^–2^ for HER, and overpotentials of 205, 230 and 430 mV at 10, 100 and 1000 mA cm^–2^ for OER, respectively. Moreover, it could afford a stable current density of 10 mA cm^–2^ for overall water splitting at 1.51 V in 1.0 M KOH with long-term durability (100 h). This cell voltage is among the best reported values for bifunctional electrocatalysts. The results of theoretical calculations demonstrate that P-NiFe displays optimized adsorption energies for both HER and OER intermediates at the nickel active sites, thus dramatically enhancing its electrocatalytic activity.

## Abstract 2

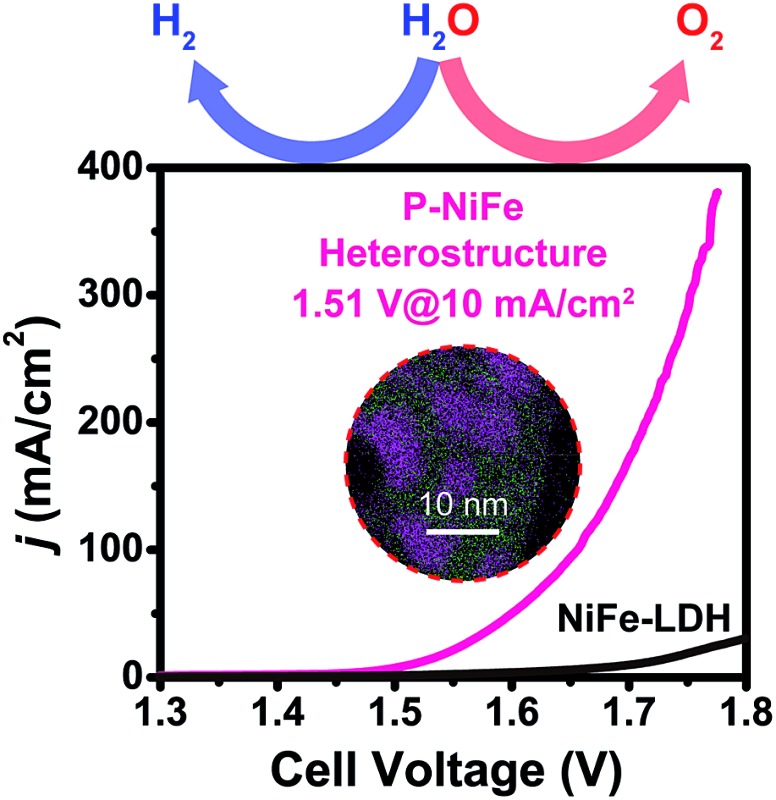
Ni_2_P@FePO*_x_* has been synthesized *via* extraction and selective phosphorization of nickel in NiFe-LDH, and then conversed to a new heterostructure of Ni_2_P@NiFe hydroxide (P-NiFe) during water splitting. P-NiFe can act as an efficient bifunctional electrocatalyst for overall water splitting.

## Introduction

To address energy and environmental problems, hydrogen fuel has been regarded as a promising alternative to fossil fuels for its high energy density and cleanness.^1^ Electrochemical water splitting has been regarded as a promising method for the production of hydrogen, using electric energy coming from intermittent solar energy and wind energy sources.^2^ However, water splitting is a thermodynamically uphill process, accompanied by high overpotentials (*η*) for both HER and OER, which requires efficient and stable electrocatalysts to significantly reduce the overpotentials.^3^ As is well known, Pt-based materials and iridium oxides are the state-of-the-art electrocatalysts for HER and OER, respectively,^4^ while their high cost and scarcity seriously limit their large-scale application. Therefore, there has been growing interest in developing earth-abundant and cheap electrocatalysts for water splitting, and great progress has been achieved over past years. It has been found that transition-metal sulfides,^5^ carbides,^6^ selenides^7^ and phosphides^8^ can dramatically reduce the overpotentials for HER in acidic media, and the transition-metal oxides/hydroxides^9^ exhibit remarkable electrocatalytic activity for OER in alkaline solution. However, it remains a great challenge to pair the HER and OER electrocatalysts in an integrated electrolyzer for efficient overall water splitting, due to the mismatch between their best working conditions. Bifunctional electrocatalysts to facilitate both HER and OER in the same electrolyte are highly appealing, as this can lower the total cost by avoiding the preparation of different catalysts, and make water splitting feasible for practical use.3b,4a,^10^ Nonetheless, only a few bifunctional electrocatalysts display competitive catalytic activity to that of a Pt–IrO_*x*_-coupled electrolyzer that can reduce the overall water splitting cell voltage to near 1.5 V at a current density of 103b,4a,10c,^10^ or 20 mA cm^–2^.10*e* In this context, the design and synthesis of efficient bifunctional electrocatalysts for overall water splitting in the same electrolyte still remain a great challenge.

Previous studies have revealed that binary/ternary metal hydroxides/oxides exhibit higher electrocatalytic performance for OER than their unary counterparts,^11^ possibly due to the optimized binding energies for OER intermediates (*OH, *O, and *OOH) on the catalyst surface,9*c* and/or enhanced charge transferability of the catalysts in the presence of doping elements. NiFe-layered double hydroxide (NiFe-LDH) based nanomaterials, with binary metal centers evenly mixed on a molecular level, have shown high performance on OER, with low overpotentials of 210–350 mV at 10 mA cm^–2^.9a,10e,^12^ However, when they were used as a bifunctional electrocatalyst for overall water splitting, the cell voltage was as high as 1.7 V at a current density of 10 mA cm^–2^ in 1.0 M KOH,12*b* due to their higher overpotential for HER. Accordingly, decreasing the overpotential for HER will make NiFe-LDH into an efficient bifunctional electrocatalyst for overall water splitting.

Recently, transition-metal phosphides have also been demonstrated as efficient electrocatalysts for HER in an alkaline medium,8h,8i,^13^ besides their high HER catalytic activity in acidic media.8a,8d,8g,^14^ In addition, it has been demonstrated that bimetallic phosphide NiCoP displays higher HER activity than monometallic phosphide Ni_2_P, due to its optimized H binding energy of nearly zero (close to that of Pt) on the surface of NiCoP, which is beneficial for the reversible adsorption and desorption of H.13*a* We presume that the HER activity could be improved by the phosphorization of NiFe-LDH to get a bimetallic phosphide NiFeP, and combine it with a highly efficient NiFe-LDH OER electrocatalyst to construct an NiFeP/NiFe-LDH-coupled electrolyzer, when the cell voltage would be dramatically decreased. Unexpectedly, an Ni_2_P@FePO_*x*_ heterostructure was obtained during the phosphorization of NiFe-LDH, in which Ni was selectively phosphorized and extracted from NiFe-LDH. Ni_2_P@FePO_*x*_ further evolved to Ni_2_P@NiFe hydroxide (P-NiFe) during water splitting, which shows a much lower overpotential of 75 mV than that of 230 mV for NiFe-LDH at 10 mA cm^–2^ for HER in 1.0 M KOH solution. More interestingly, P-NiFe also shows a better OER performance than NiFe-LDH in 1.0 M KOH solution, with overpotentials of 205, 230 and 430 mV at current densities of 10, 100 and 1000 mA cm^–2^, respectively, much lower than the corresponding values of 250, 280, and 560 mV for NiFe-LDH at current densities of 10, 100 and 1000 mA cm^–2^, respectively. Therefore, a P-NiFe heterostructure can serve as an efficient bifunctional electrocatalyst for an alkaline electrolyzer, generating a cell voltage of only 1.51 V at 10 mA cm^–2^ in 1.0 M KOH solution, even better than the combination of the state-of-the-art IrO_2_ and Pt/C as benchmark electrocatalysts. Though a number of NiFe-based metal hydroxides/oxides9a,12b–f,12h and transition-metal phosphides8a,8e,8f,8i,^15^ have been reported as electrocatalysts for OER and HER, to our knowledge, the selective extraction and phosphorization of a single metal from mixed metal hydroxides/oxides to form a heterostructure has not been documented so far. The *in situ* transformed Ni_2_P@NiFe hydroxide heterostructure not only enhances its conductivity due to the existence of metallic Ni_2_P, but also optimizes the adsorption energies for both HER and OER intermediates at the nickel active sites on its surface, thus dramatically enhancing its electrocatalytic activity for both OER and HER.

## Experimental section

### Materials

Ni(NO_3_)_2_·6H_2_O (99.9985% metals basis, Alfa), Fe(NO_3_)_3_·9H_2_O, (98+% metals basis, Alfa), urea (99.999%, Aladdin), NaH_2_PO_2_ (99.0%, Aladdin), IrO_2_ (99.99% metals basis, Alfa), 20% Pt on Vulcan XC72 (20% Pt/C, Sigma-Aldrich), Nafion (5 wt%, Sigma-Aldrich), nickel foam (>99.5%, 1.0 mm thick, Taiyuan Yingze Lizhiyuan Battery) and other materials were obtained from commercial suppliers and used without further purification, unless otherwise noted.

### Characterization

Scanning electron microscopy (SEM) and energy dispersive X-ray spectroscopy (EDS) results were collected by a field emission scanning electron microscope (FEI, Quanta 400). High-resolution transmission electron microscope (HRTEM) images, quantitative EDS data and electron energy loss spectroscopy (EELS) elemental mapping were taken on an aberration-corrected scanning transmission electron microscope (STEM, FEI Titan Cubed Themis G2 300 at 200 and 300 kV). The powder X-ray diffraction measurements were performed on a D8 ADVANCE X-ray Diffractometer. The X-ray photoelectron spectroscopy (XPS) data was collected on an ESCA Lab250 instrument. Raman spectra were carried out on a Renishaw in Via Laser Micro-Raman Spectrometer (Horiba-Jobin-Yvon T64000 instrument) using a 514 nm laser. Electrocatalytic properties were studied with a standard three-electrode system controlled by a CHI760E electrochemical workstation. An Ag/AgCl (in 3 M KCl solution) electrode and a carbon rod were used as the reference electrode and the counter electrode, respectively. The linear sweep voltammetry curves were recorded at a scan rate of 5 mV s^–1^ in 1.0 M KOH solution. The electrochemical impedance spectroscopy (EIS) measurements were conducted over a frequency range from 10 kHz to 100 kHz. The analysis of the gas product was operated on a gas chromatograph (Agilent 7820A-GC, molecular sieve columns, thermal-conductivity detector, TCD).

### Preparation

#### Preparation of NiFe-LDH@NF

The NiFe-LDH was prepared according to a modified literature method.12*b* Ni(NO_3_)_2_·6H_2_O (0.5 mmol), Fe(NO_3_)_3_·9H_2_O (0.5 mmol) and urea (2.5 mmol) were mixed in 40 mL of deionized water. The resulting solution was poured into a 50 mL autoclave with a piece of 1 × 2 cm NF leaning against the wall. The growth was carried out at 120 °C in an electric oven for 12 h. After natural cooling to room temperature, the NiFe-LDH sample coated on NF was collected, washed with deionized water, and then blown dry under a stream of compressed air.

#### Preparation of Ni(OH)_2_@NF

Ni(OH)_2_@NF was prepared by a similar procedure to the synthesis of NiFe-LDH@NF without adding Fe(NO_3_)_3_·9H_2_O, and 1 mmol Ni(NO_3_)_2_·6H_2_O was used in the synthesis process.

#### Preparation of P-NiFe@NF and P-Ni@NF

250 mg of NaH_2_PO_2_ was placed at the upstream side of a tube furnace and one piece of 1 × 2 cm NiFe-LDH@NF was located at the downstream side of the furnace. Then, the furnace was heated to 573 K with a heating rate of 2 K min^–1^ under an Ar flow, and maintained at 573 K for 3 h. After natural cooling to room temperature, Ni_2_P@FePO_*x*_ was obtained, which was *in situ* transformed to Ni_2_P@NiFe hydroxide (P-NiFe@NF electrode) during water splitting. The Ni_2_P@Ni(OH)_2_ (P-Ni@NF) was prepared by a similar procedure with P-NiFe/NF using Ni(OH)_2_@NF instead of NiFe-LDH@NF. The amount of Ni_2_P@FePO_*x*_ or Ni_2_P loading on NF, which was determined by the weight difference of NF before and after material growth, is approximately 1.0 mg cm^–2^.

#### Preparation of 20% Pt/C@NF and IrO_2_@NF

10 mg of 20% Pt/C (or IrO_2_) and 50 μL of Nafion (5 wt%) were dispersed in 1 mL of ethanol with sonication for at least 30 min to generate a homogeneous ink. Then 150 μL of the ink was drop-casted onto a NF, and the solvent was evaporated at room temperature overnight. The amount of loading is approximately 1.0 mg cm^–2^.

### Estimation of effective electro-chemical surface area (ECSA)

To evaluate the ECSA, cyclic voltammetry (CV) was carried out to probe the electrochemical double-layer capacitance (*C*_dl_) of various samples in the non-faradaic region from CV in a quiescent solution. This non-faradaic region is typically a 0.1 V window with the open circuit potential as the middle point. All measured current in this region is assumed to be due to double-layer charging. By plotting the current density at 0.92 V *vs.* RHE against the scan rate, a linear trend was observed. The linear slope, equivalent to twice of the double-layer capacitance *C*_dl_, was used to represent the ECSA.

### Estimation of Faraday efficiency

The detection of hydrogen and oxygen was performed in a one-compartment, two-electrode cell with stirring. Two P-NiFe@NF electrodes (1 × 1 cm) were used as the working electrode and the counter electrode, respectively. Before the detection of the gas product, the cell was firmly sealed to be gas-tight and subsequently purged with argon for 20 min. Before and after the electrolysis at a current density of 10 mA cm^–2^, the gas products were analyzed by gas chromatography.

### DFT calculation

According to previous studies,^16^ the Gibbs free energy of each elementary step of OER or HER were calculated as follows:Δ*G* = Δ*E* + ΔZPE – *T*Δ*S*where Δ*E* is the reaction energy calculated by using the spin polarization density functional theory (DFT) method. ΔZPE and Δ*S* are the changes in zero point energies and entropy during the reaction, respectively. In the case of HER calculations, as the vibrational entropy of H* in the adsorbed state is small, the entropy of adsorption of 1/2H_2_ is Δ*S*_H_ ≈ –0.5*S*_0H_2__, where *S*_0H_2__ is the entropy of H_2_ in the gas phase under the standard conditions. Therefore the overall corrections were taken as in^17^Δ*G*_H*_ = Δ*E*_H*_ + 0.24 eV

Based on the structural characterizations, the periodical surface models of the heterostructures were constructed by using the crystal structures of Ni_2_P (inner layer) and Ni/Fe hydroxide (outer layer), and the latter were all set to be charge neutral by tuning the numbers of H atoms on the oxygen. The higher index 
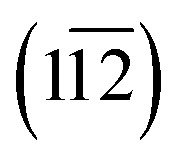
 facet of Fe/Ni(OH)_*x*_, containing under-coordinated metal sites similar to a step or edge, was adopted as it possesses higher OER activity.^18^ A vacuum slab of 15 Å is used to separate the layer from its periodic images. All the geometrical optimizations and energy calculations were performed by the Dmol^3^ module in Materials Studio 5.5. The widely used generalized gradient approximation (GGA) with the Perdew–Burke–Ernzerhof (PBE) function and the double numerical plus polarization (DNP) basis set were used for all the non-metal atoms. An accurate DFT semi-core pseudopots (DSPP) was employed for metal atoms, and thermal smearing was applied to the orbital occupation to speed up convergence. For all the DFT calculations, the energy, gradient and displacement convergence criteria were set at 2 × 10^–5^ Ha, 4 × 10^–3^ Å and 5 × 10^–3^ Å, respectively.

## Results and discussion

### Synthesis and characterization

**Scheme 1:**
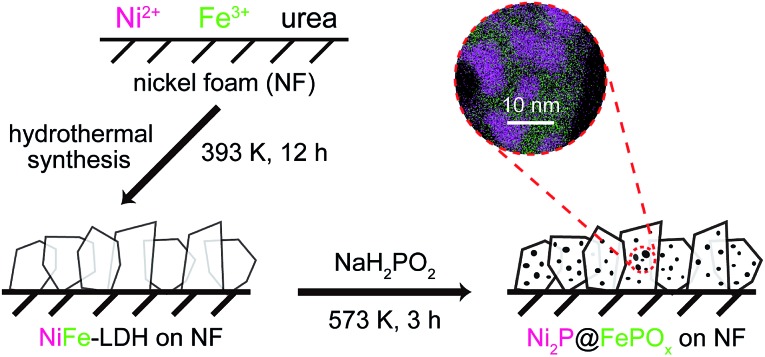
The procedure for the synthesis of Ni_2_P@FePO_*x*_ nanosheets.

The Ni_2_P@FePO_*x*_ heterostructure was prepared by the phosphorization of highly dense, vertically aligned NiFe-LDH nanosheets on an NF (nickel foam) substrate (Scheme 1). At first, the vertically aligned NiFe-LDH nanosheets were uniformly grown on the NF substrate by a hydrothermal method, as observed by SEM (Fig. S1†), and its phase purity was confirmed by the X-ray diffraction (XRD) measurement (Fig. S2†). Subsequently, the NiFe-LDH nanosheets were phosphorized using NaH_2_PO_2_ as a P source. With plenty of parallel experiments, it was found that the phosphorization temperature plays a key role during the formation of Ni_2_P@FePO_*x*_ nanosheets. Based on the decomposition temperature of NaH_2_PO_2_ (above 473 K),^19^ the phosphorization temperatures were selected from 473 to 773 K, with a controlled reaction time scale of 3 h. As shown in Fig. S3a,† the morphology of the sample prepared at 473 K barely changed compared to that of the original NiFe-LDH nanosheets, and a large amount of NaH_2_PO_2_ was found to be unreacted, suggesting that the phosphorization temperature of 473 K is too low. The nanosheet-like morphology of the sample obtained at 573 K can be retained, though the sheet edges become thicker compared to those of the original NiFe-LDH nanosheets (Fig. S3b†). Further increasing the phosphorization temperatures to 673 and 773 K will destroy the morphology of the nanosheets (Fig. S3c and d†). On the other hand, the phosphorization time was also found to play an important role during the formation of Ni_2_P@FePO_*x*_ nanosheets, and 3 h was found to be an ideal phosphorization time for the preparation of Ni_2_P@FePO_*x*_ nanosheets (Fig. S4b†). Further increasing the phosphorization time will destroy the morphology of the nanosheets (Fig. S4c†). Therefore, the optimized phosphorization temperature and time are 573 K and 3 h, respectively, and the Ni_2_P@FePO_*x*_ nanosheets synthesized under such optimized conditions were used as an electrocatalyst for further investigation (see Scheme 1). The direct integration of an Ni_2_P@FePO_*x*_ electrocatalyst on NF not only provides conductive support and a 3D macroporous feature which can be used directly as an electrode, but also avoids the use of expensive electrodes and the requirement for extra glues to stick the catalysts onto the surface of such electrodes, which is beneficial for a commercial water electrolyzer.

**Fig. 1:**
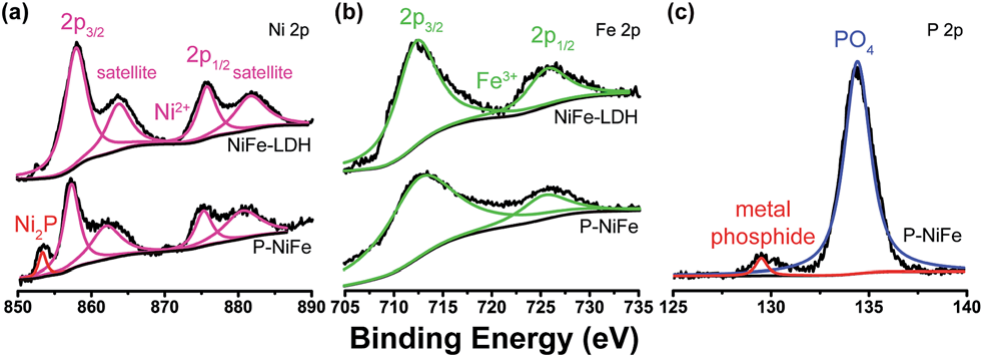
The XPS patterns of as-prepared Ni_2_P@FePO_*x*_ sample, showing the signals of (a) Ni 2p, (b) Fe 2p and (c) P 2p.

X-ray photoelectron spectroscopy (XPS) was used to determine the chemical composition on the surface of Ni_2_P@FePO_*x*_. As shown in Fig. 1a, the XPS of as-prepared Ni_2_P@FePO_*x*_ material exhibits a binding energy peak at 853.5 eV, and this peak is absent in the XPS pattern of NiFe-LDH, which can be attributed to metallic nickel in the nickel phosphide (Ni_2_P).15*b* The presence of Ni_2_P in the Ni_2_P@FePO_*x*_ sample can be further confirmed by the X-ray diffraction (XRD) measurements, which show the characteristic diffraction peaks of Ni_2_P (Fig. S5†). The XPS patterns also display the binding energies of Ni 2p_3/2_ and Ni 2p_1/2_ peaks located at 857.2 and 875.2 eV, respectively (Fig. 1a). These values are in good agreement with those of Ni^2+^ in NiFe-LDH and other nickel oxides or phosphates,8f,15a which can be attributed to the surface oxidation of Ni_2_P. The peaks of Fe 2p_3/2_ and Fe 2p_1/2_ in the P-NiFe sample appear at 712.5 and 725.5 eV, respectively (Fig. 1b), demonstrating the presence of the Fe^3+^ state.^14^ In addition, the XPS pattern of P 2p shows the existence of both metal phosphide (129.7 eV)8e,15c and metal phosphate (134.3 eV, Fig. 1c).13b,15c The binding energy of 853.5 eV is slightly higher than that of metallic Ni (852.6 eV),^20^ while the binding energy of 129.7 eV is slightly lower than that of elemental P (130.0 eV),^20^ indicating that the Ni and P are partially positive and negatively charged in Ni_2_P, respectively. All the above results indicate the co-existence of Ni_2_P, Ni^2+^, Fe^3+^ and metal phosphate species in the Ni_2_P@FePO_*x*_ sample.

**Fig. 2:**
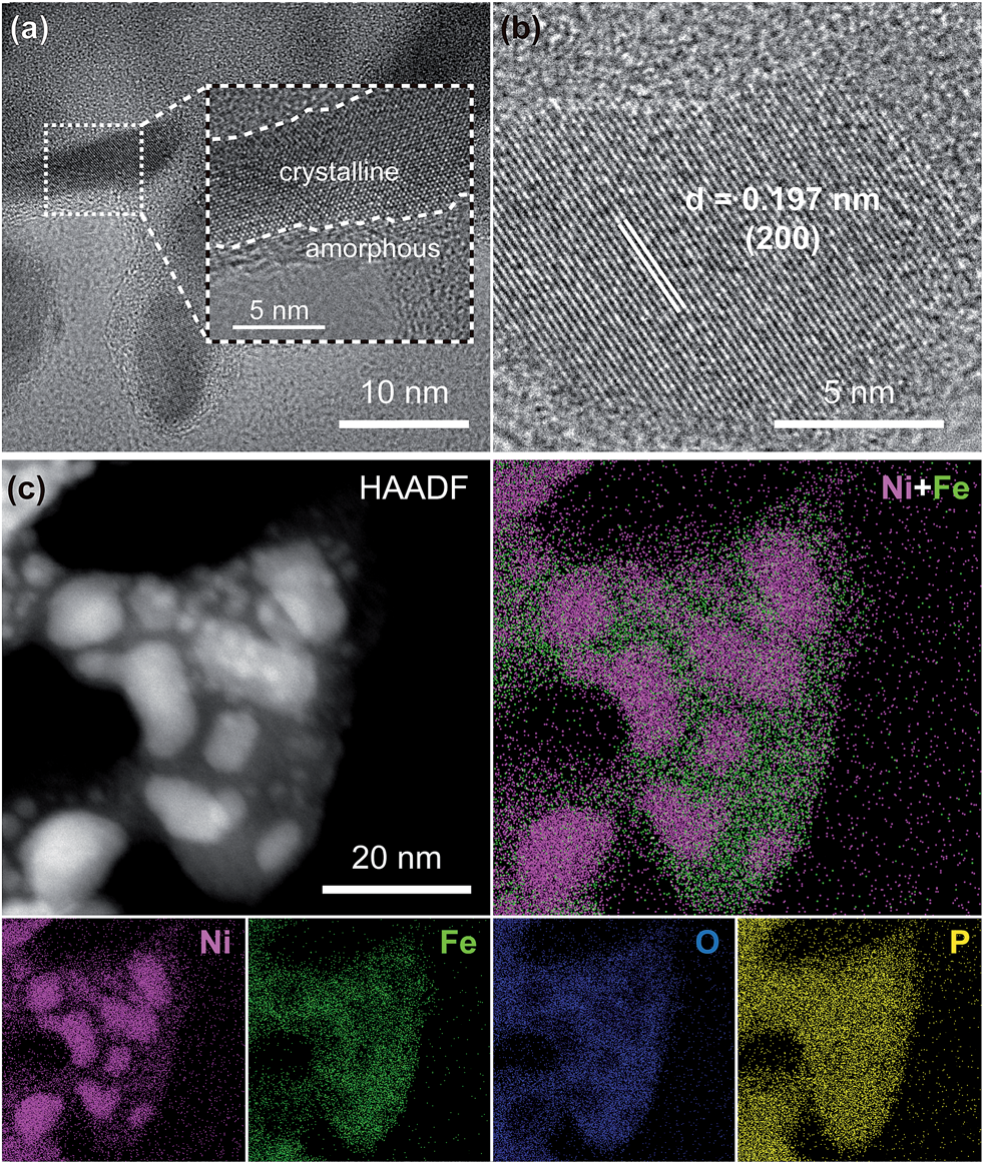
(a) TEM image, (b) HRTEM image, (c) HAADF-STEM image and EDS elemental mapping images for the Ni_2_P@FePO_*x*_ sample.

To further analyze the morphology and composition of the as-prepared Ni_2_P@FePO_*x*_ sample, a scanning transmission electron microscope (STEM) with a high-angle annular dark field (HAADF) mode was utilized. As shown in Fig. 2a and S6,† the high-resolution transmission electron microscope (HRTEM) images show crystalline nanoparticles (10–20 nm) embedded in an amorphous substrate. The lattice fringes of the crystalline nanoparticles can be clearly observed (Fig. 2b), where the (200) crystal facet with a *d*-spacing of 0.197 nm is in agreement with the crystal phase of Ni_2_P (JCPDS no. 74-1385), further demonstrating the formation of crystalline Ni_2_P nanoparticles in Ni_2_P@FePO_*x*_. In the substrate, no lattice fringes can be observed, suggesting the amorphous nature of the substrate. As shown in the energy dispersive X-ray spectroscopy (EDS) elemental mapping images (Fig. 2c), the majority of the Ni element is distributed in the crystalline Ni_2_P nanoparticles, while the Fe and O elements are mainly dispersed in the amorphous substrate. Additionally, the P element is distributed over the Ni_2_P@FePO_*x*_ sample homogeneously. These observations indicate that the crystalline nanoparticles are mainly comprised of Ni_2_P, and the amorphous substrate is most likely to be iron phosphate (FePO_*x*_). The presence of Fe_2_O_3_ species in the amorphous substrate can be excluded by comparison of the results of electron energy loss spectroscopy (EELS) for P-NiFe with those of the Fe_2_O_3_ sample (Fig. S7†).^21^ The results of EDS measurement for the Ni_2_P@FePO_*x*_ sample reveal that the ratio of Ni : P : Fe : O is approximately 2 : 2 : 1 : 4 (Fig. S8†), indicating that the ratio of Ni_2_P and FePO_*x*_ (*x* ≈ 4)^22^ in Ni_2_P@FePO_*x*_ is approximately 1 : 1. All the above observations clearly demonstrate that the Ni_2_P@FePO_*x*_ sample consists of crystalline Ni_2_P nanoparticles embedded in amorphous iron phosphate, forming an Ni_2_P@FePO_*x*_ heterostructure. The formation of Ni_2_P nanoparticles suggests that Ni was selectively phosphorized and extracted from NiFe-LDH during the phosphorization process. This is possibly because the Ni^2+^ in NiO and Ni(OH)_2_ could be easily phosphorized to form Ni_2_P by substantial substitution of O atoms by P atoms.8*f* In contrast, the iron hydroxide was transformed to iron phosphate rather than iron phosphide, presumably due to the high affinity of Fe^3+^ towards O, which may hinder the substitution of O atoms by P atoms.^21^ The extraction of Ni from NiFe-LDH during the phosphorization process will produce a large amount of defects in the synthesized Ni_2_P@FePO_*x*_ heterostructure, which will provide more active sites for HER and OER, giving rise to enhanced HER and OER activity.

### Electrocatalytic HER

**Fig. 3:**
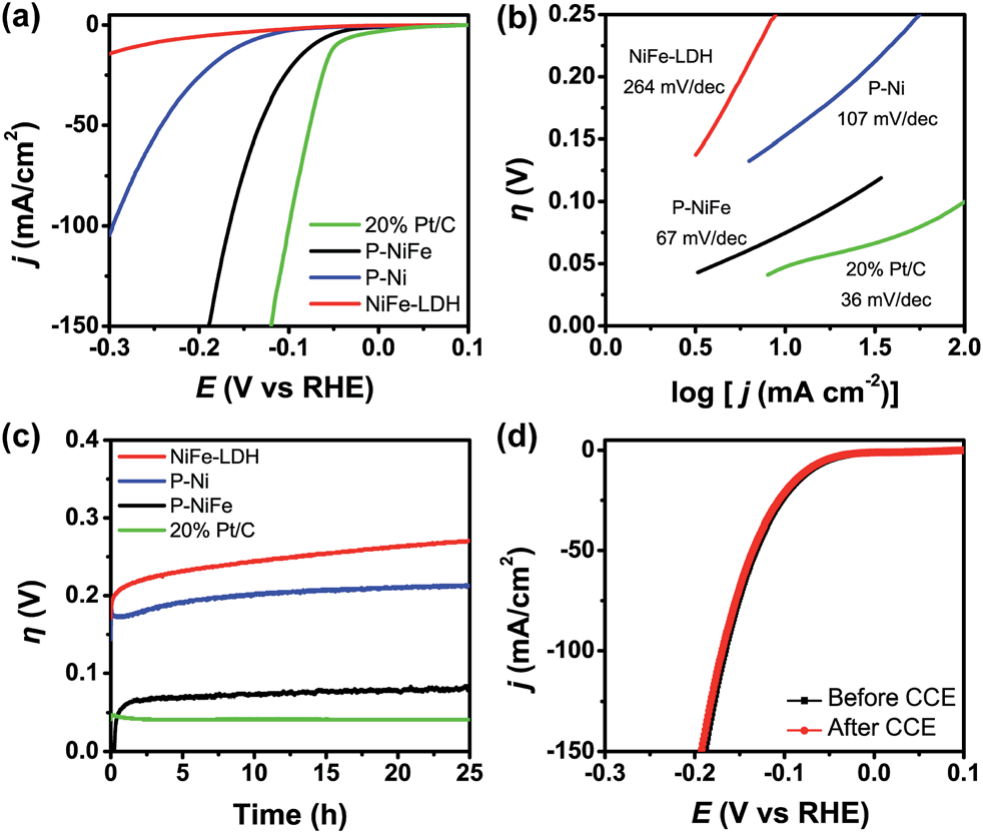
(a) IR-corrected linear sweep voltammetry (LSV) curves, with a scan rate of 5 mV s^–1^. (b) Tafel plots and (c) current density traces of CCE at 10 mA cm^–2^ for HER in 1.0 M KOH. (d) LSV curves for P-NiFe before (black line) and after (red line) CCE for HER at 10 mA cm^–2^ for 25 h. Catalysts: P-NiFe (black), NiFe-LDH (red), P-Ni (blue) and 20% Pt/C (green).

The electrocatalytic activity of as-prepared Ni_2_P@FePO_*x*_ for HER was investigated in 1.0 M KOH aqueous solution, and the results indicate that Ni_2_P@FePO_*x*_ was converted to Ni_2_P@NiFe hydroxide (P-NiFe) during HER as a real electrocatalyst (see below). For comparison, the electrocatalytic performances of NiFe-LDH, P-Ni (obtained by phosphorization of nickel hydroxides) and commercial 20% Pt/C coated on NF were also investigated under the same conditions. It is exciting to note that P-NiFe exhibits high catalytic activity for HER in alkaline aqueous solution, with an overpotential of 75 mV at 10 mA cm^–2^ (Fig. 3a). This value is much lower than those of P-Ni (190 mV) and NiFe-LDH (230 mV) under the same conditions, and is comparable to that of commercial 20% Pt/C (45 mV). Moreover, the Tafel slope of P-NiFe (67 mV dec^–1^) is also lower than those of P-Ni (107 mV dec^–1^) and NiFe-LDH (264 mV dec^–1^; Fig. 3b), indicating the rapid HER catalytic rate of P-NiFe. Besides the high catalytic efficiency for HER, the P-NiFe catalyst also exhibits impressive robustness during a 25 h controlled-current electrolysis (CCE) at 10 mA cm^–2^, with a stable overpotential of 75 mV (Fig. 3c). Meanwhile, the LSV curves before and after 25 h of CCE further confirm its high stability during the HER (Fig. 3d). The bare NF shows poorer activity for HER than that of P-NiFe under identical conditions (Fig. S9a†).

After the CCE for HER at 10 mA cm^–2^ for 25 h, HRTEM (Fig. S10†) and elemental mapping images (Fig. S11†) of the generated sample indicate that there was no obvious change in the morphology of the crystalline Ni_2_P nanoparticles, while the amorphous iron phosphate was transformed to crystalline Fe(OH)_3_ in 1.0 M KOH, and this was confirmed by XRD measurement (Fig. S12†), which demonstrates that both crystalline Ni_2_P and Fe(OH)_3_ coexist in P-NiFe after the CCE for HER. However, the low-energy peaks of 853.5 and 129.7 eV belonging to Ni_2_P disappeared in the XPS patterns for the P-NiFe sample after CCE (Fig. S13†), and only the peaks (857.1 and 875.2 eV) attributed to Ni^2+^ were observed, indicating that the surface of Ni_2_P was oxidized into Ni(OH)_2_ in alkaline solution, and the Ni(OH)_2_ formed *in situ* at the surface of the Ni_2_P nanoparticles prevents further oxidation of Ni_2_P. However, the peak of 133.2 eV belonging to P 2p can be observed (Fig. S13c†), indicating that the Ni(OH)_2_ layer formed *in situ* at the surface of the Ni_2_P is very thin. The XPS patterns for the P-NiFe sample after the LSV measurement and before CCE (Fig. S14†) is similar to those after CCE, indicating that the surface oxidation of Ni_2_P into Ni(OH)_2_ occurs rapidly in solution. The surface oxidation of Ni_2_P into Ni(OH)_2_ and the transformation of amorphous iron phosphate into Fe(OH)_3_ in Ni_2_P@FePO_*x*_ after CCE were further confirmed by the results of Raman spectra measurements (Fig. S15†), which show very weak characteristic peaks of NiFe hydroxide, indicating the presence of trace amounts of NiFe hydroxide on the surface of the Ni_2_P nanoparticles.13c,^23^ The results of electrochemical impedance spectroscopy (EIS) measurements indicate that the conductivity of P-NiFe after HER is dramatically increased compared to that of NiFe-LDH after HER (Fig. S16a†), which can be attributed to the existence of highly conductive metallic Ni_2_P nanoparticles in P-NiFe. The results of effective electro-chemical surface area (ECSA) measurements (Fig. S17a†) indicate that number of active sites in P-NiFe after CCE for HER (29.1 mF cm^–2^) is 17-fold higher than those of NiFe-LDH after CCE for HER (1.7 mF cm^–2^). The increased number of active sites in P-NiFe could be ascribed to the extraction of Ni from NiFe-LDH during the phosphorization process, which generates a large amount of defects to act as catalytically active sites. Indeed, the surface of P-NiFe after HER becomes rougher compared with that of as-synthesized Ni_2_P@FePO_*x*_ (Fig. S18†), indicating that more active sites were exposed.

**Fig. 4:**
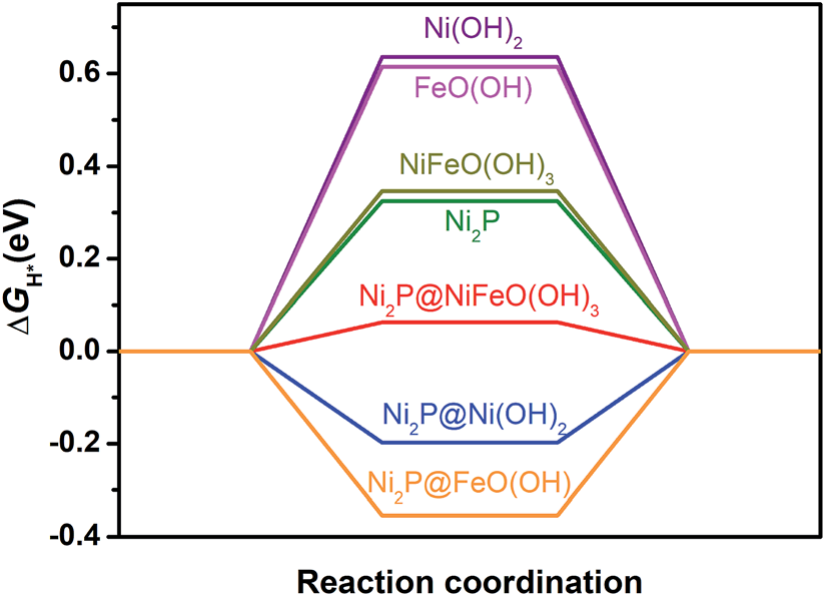
Free energy of H* adsorption on different catalysts by DFT calculations.

To reveal the origin of the enhanced HER activity of the P-NiFe, density functional theory (DFT) calculations were carried out. Generally, hydrogen evolution activity is closely related with the Gibbs free energy of hydrogen adsorption (Δ*G*_H*_) on the surfaces of catalysts in both acid and alkaline conditions, so |Δ*G*_H*_| is one of the key catalytic descriptors for theoretical prediction of HER activity, which is usually proposed as having an optimal value close to zero.^24^ In this context, we investigated seven model structures by the calculation of the free energies for H adsorption on the surfaces of different catalytic sites of Ni_2_P, Ni(OH)_2_, FeO(OH), Ni_2_P@Ni(OH)_2_, Ni_2_P@FeO(OH), NiFeO(OH)_3_ (corresponding to NiFe-LDH), and Ni_2_P@NiFeO(OH)_3_ (corresponding to P-NiFe) (Fig. S19†), and the lowest free energy for each structure is given in Fig. 4. From Fig. 4 it can be found that the single-metal LDH of Ni(OH)_2_ and FeO(OH) have the largest Δ*G*_H*_ of over 0.60 eV. After doping with Fe, the Δ*G*_H*_ of the mixed-metal NiFeO(OH)_3_ is reduced to 0.49 eV. By compositing with Ni_2_P, all the |Δ*G*_H*_| values of the LDH species can be greatly decreased, which is consistent with our experimental results, demonstrating the formation of a heterostructure of LDH and that Ni_2_P can really boost the hydrogen evolution activity. Among them, the mixed-metal heterostructure of Ni_2_P@NiFeO(OH)_3_ exhibits the lowest Δ*G*_H*_ value of 0.06 eV at the catalytic site of Ni (Fig. S19†), being closer to the ideal value of zero. Thus, it shows that the highest HER performance benefits from the synergistic electron effect after the formation of the Ni_2_P@NiFeO(OH)_3_ heterostructure.

### Electrocatalytic OER

**Fig. 5:**
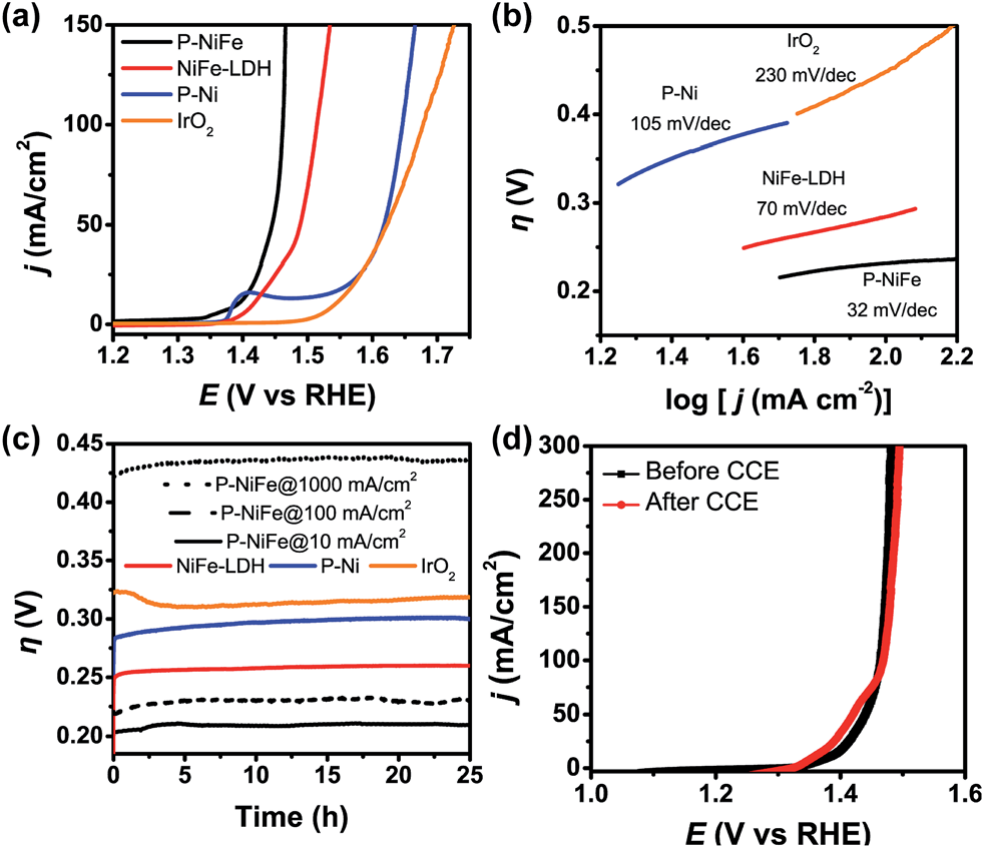
(a) IR-corrected LSV curves, with a scan rate of 5 mV s^–1^. (b) Tafel plots for OER in 1.0 M KOH and (c) current density traces of CCE at 10 mA cm^–2^ (as well as 100 and 1000 mA cm^–2^ for P-NiFe, dashed line). (d) LSV curves of P-NiFe before (black square) and after (red circle) CCE for OER at 10 mA cm^–2^ for 25 h. Catalysts: P-NiFe (black), NiFe-LDH (red), P-Ni (blue) and IrO_2_ (orange).

Electrocatalytic performance of the Ni_2_P@FePO_*x*_ catalyst for OER was also evaluated in 1.0 M KOH solution at a scan rate of 5 mV s^–1^, and the results indicate that Ni_2_P@FePO_*x*_ was also converted to Ni_2_P@NiFe hydroxide (P-NiFe) during OER as a real electrocatalyst (see below). For comparison, the electrocatalytic performances of NiFe-LDH, P-Ni and commercial IrO_2_ coated on NF were also investigated under the same conditions. As shown in Fig. 5a, P-NiFe exhibits a much higher current density at a given potential of 1.45 V in comparison to those of other catalysts. To avoid the interference of Ni^III/II^ oxidation waves at approximately 1.4 V, CCE was used to determine the overpotentials for OER of these catalysts at a current density of 10 mA cm^–2^. As shown in Fig. 5c, P-NiFe displays a stable overpotential of 205 mV at 10 mA cm^–2^ during a 25 h CCE. This value is much lower than those of NiFe-LDH (250 mV), P-Ni (290 mV) and IrO_2_ (320 mV), indicating that the OER catalytic activity is also enhanced after the formation of a P-NiFe heterostructure. In addition, the Tafel slope of P-NiFe (32 mV dec^–1^) is smaller than those of NiFe-LDH (70 mV dec^–1^), P-Ni (105 mV dec^–1^) and IrO_2_ (230 mV dec^–1^, see Fig. 5b), indicating its fast catalytic rate and favorable OER kinetics. The high stable catalytic performance of P-NiFe was further confirmed by the LSV measurements, in which the LSV curves before and after 25 h of CCE at 10 mA cm^–2^ are almost identical (Fig. 5d).

For commercial hydrogen production by electrocatalytic water splitting, the development of non-precious OER electrocatalysts that can work with long-term stability at large current densities and low overpotentials is highly desirable. For the P-NiFe electrocatalyst, it only requires very low OER overpotentials of 230 and 430 mV to achieve large current densities of 100 and 1000 mA cm^–2^, respectively, and P-NiFe exhibits impressive robustness for at least 25 h during CCE at a large current density of 1000 mA cm^–2^ in 1.0 M KOH solution (Fig. 5c), which exhibits much better performance than NiFe-LDH, in which NiFe-LDH shows an overpotential of 550 mV to achieve a current density of 1000 mA cm^–2^, with the overpotential increasing to 650 mV after 25 h of CCE (Fig. S20†). To our knowledge, these values are among the lowest overpotentials of reported OER electrocatalysts for the corresponding current densities (see Table S1†). It should be mentioned that bare NF shows much lower activity for OER than that of P-NiFe under the same conditions (Fig. S9b†), indicating that the high OER activity originates from the P-NiFe catalyst rather than from the NF.

All the above results reveal that the OER catalytic activity of P-NiFe is also much enhanced compared to that of NiFe-LDH. In order to gain an in-depth understanding of the origin of the high catalytic activity of P-NiFe, a series of characterizations were performed on the P-NiFe catalyst after 25 h of CCE for OER. HRTEM (Fig. S21†) and elemental mapping images (Fig. S22†) indicate that the original amorphous iron phosphate in Ni_2_P@FePO_*x*_ also transformed into crystalline Fe(OH)_3_ during CCE in basic solution, while the Ni_2_P nanoparticles still exist in the heterostructure. These results are further confirmed by the XRD measurements, where the characteristic diffraction peaks of Ni_2_P and Fe(OH)_3_ were all detected (Fig. S23†). However, the XPS results show the disappearance of the peaks of P and the low-energy peak of nickel (853.5 eV), as well as a shift toward higher binding energy for Ni (from 857.2 to 857.8 eV) in P-NiFe after CCE (Fig. S24†), indicating the surface oxidation of Ni_2_P to form nickel hydroxide during OER.8f,15a,15b The XPS patterns for the P-NiFe sample after the LSV measurement and before CCE (Fig. S25†) are similar to those after CCE, indicating that the surface oxidation of Ni_2_P into Ni(OH)_2_ occurs rapidly during OER in solution. Furthermore, after the CCE, new peaks corresponding to the NiFe hydroxide appeared in the Raman spectroscopy of P-NiFe (Fig. S15†), while no obvious peaks were observed in the Raman spectroscopy of as-synthesized Ni_2_P@FePO_*x*_. These observations indicate that the NiFe hydroxide formed on the surface of P-NiFe during the OER to serve as a real catalyst for OER,10b,13c,^23^ which also acts as a protective layer to prevent the further corrosion of Ni_2_P.15*a*

Overall, the above results demonstrate that, during the CCE for OER, the as-prepared Ni_2_P@FePO_*x*_ heterostructure was transformed into a new heterostructure comprised of NiFe hydroxide around the inner Ni_2_P nanoparticles. Such a transformation can be regarded as a self-optimization of Ni_2_P@FePO_*x*_ during the electrocatalytic process to form a more stable and more efficient catalyst of P-NiFe for OER. Similar to P-NiFe for HER, the inner Ni_2_P in P-NiFe for OER can also serve as a highly conductive, metallic support to provide a fast electron transfer pathway,13a,^25^ and this was confirmed by the results of EIS measurements, in which P-NiFe shows a smaller charge transfer impedance than that of NiFe-LDH after 25 h of CCE for OER (Fig. S16b†). In addition, the results of ECSA measurements indicate that P-NiFe possesses 5-fold more active sites than NiFe-LDH (Fig. S17b†), which can be attributed to the extraction of Ni from NiFe-LDH and the increased surface roughness of P-NiFe after CCE (Fig. S26†), resulting in more active sites and enhanced OER activity.

To further understand the origin of the enhanced OER activity of the P-NiFe, DFT calculations were performed. The results of previous DFT calculations have already demonstrated that the OER activity of WOCs based on transition metal materials is mainly driven by the energetics of the OER intermediates (*OH, *O, and *OOH, * being the adsorption site) on the catalyst surfaces, and the adsorption energy difference between O and OH is the main descriptor that affects the trends in OER activity among these materials.9*c* In this context, we investigated seven model structures to simulate the overpotentials for oxygen evolution on the surfaces of Ni_2_P, Ni(OH)_2_, FeO(OH), Ni_2_P@Ni(OH)_2_, Ni_2_P@FeO(OH), NiFeO(OH)_3_ (corresponding to NiFe-LDH), and Ni_2_P@NiFeO(OH)_3_ (corresponding to P-NiFe), respectively, on the basis of the following OER mechanism:8i,17a,^26^

1 OH^–^ → *OH + e^–^

2 OH^–^ + *OH → *O + H_2_O + e^–^

3 OH^–^ + *O → *OOH + e^–^

4 OH^–^ + *OOH → O_2_ + H_2_O + e^–^

5 *η* = max {Δ*G*_1_,Δ*G*_2_,Δ*G*_3_,Δ*G*_4_}/e – 1.23 V

The free energies of the reactions (1) to (4) are denoted as Δ*G*_1_ to Δ*G*_4_, respectively, and the overpotential *η* is defined in eqn (5). As is known, the rate-limiting step during OER for a given catalyst is the step with maximum Δ*G* value among Δ*G*_1_ to Δ*G*_4_, the lower rate-limiting energy barrier will lead to the smaller overpotential *η* (eqn (5)) and subsequently lead to higher OER activity.

**Fig. 6:**
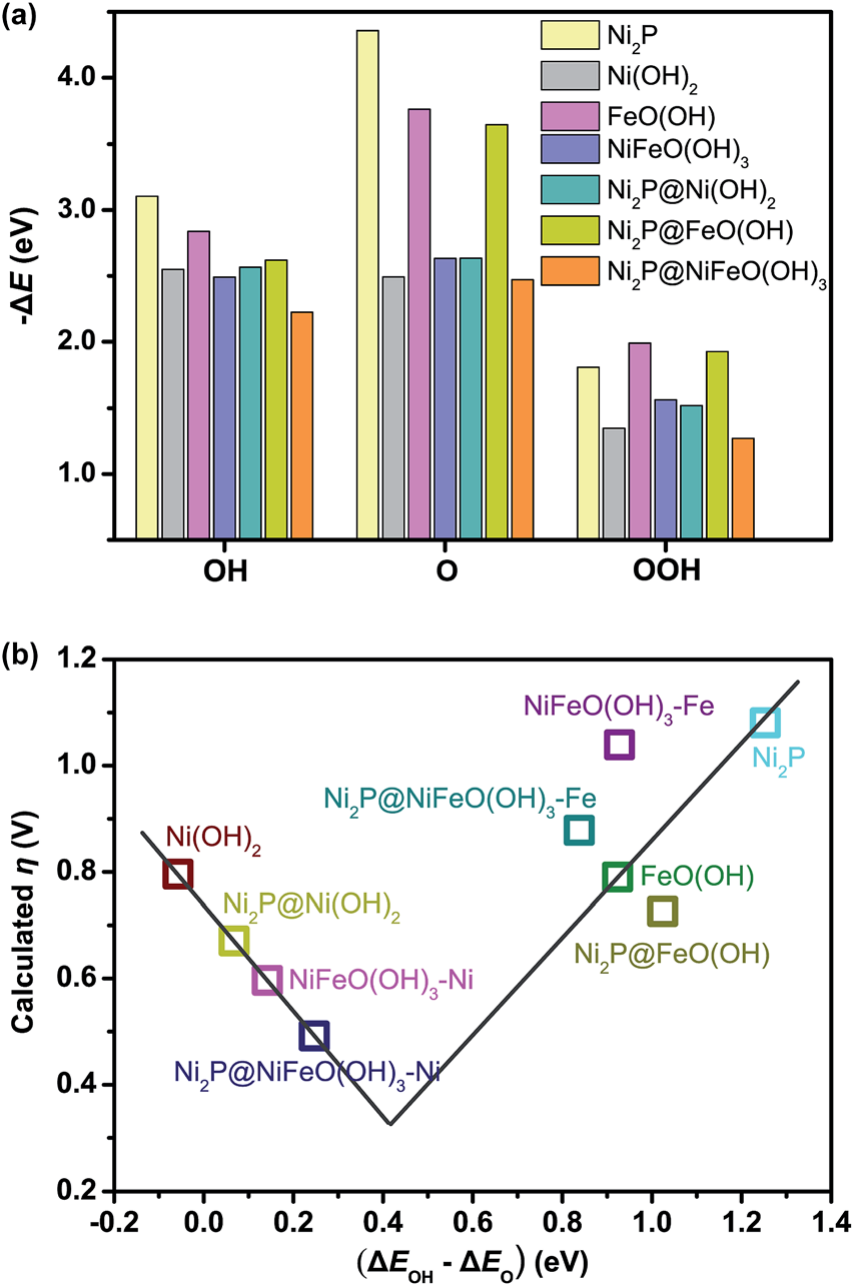
(a) Calculated binding energies of the OER intermediates, and (b) volcano-shaped plot of calculated overpotential *versus* the differences in binding energies between OH and O on the simulated surfaces of different catalysts. NiFeO(OH)_3_–Ni and Ni_2_P@NiFeO(OH)_3_–Ni represent the catalytic active sites for Ni, while NiFeO(OH)_3_–Fe and Ni_2_P@NiFeO(OH)_3_–Fe represent the catalytic active sites for Fe.

The optimized structures of the intermediates in the free-energy landscape are shown in Fig. S27.† From Fig. S27† it can be found that Ni_2_P@NiFeO(OH)_3_ displays the lowest rate-limiting energy barrier of 1.722 eV (Δ*G*_1_), with the smallest calculated *η* value of 0.49 V. For Ni_2_P, the calculated results show that the rate-limiting step is the formation of *OOH (reaction (3)) in an ideal Ni_2_P surface, with a very large overpotential (*η* = 1.08 V), due to its largest binding energy of O as well as the relatively smaller binding energy of OOH (Fig. 6a), and the larger binding energies of Ni_2_P towards OH and O may be attributed to the metallic nature of Ni. For the surface-oxidized Ni_2_P@Ni(OH)_2_ heterostructure, the rate-limiting step is the formation of *O (reaction (2)), with a smaller *η* value of 0.62 V compared to that of pure Ni(OH)_2_ (*η* = 0.80 V), this is because the relatively smaller binding energy of O on Ni(OH)_2_ has remarkably increased after combining with inner Ni_2_P in the Ni_2_P@Ni(OH)_2_ heterostructure (see Fig. S27†), as Ni_2_P possesses the largest binding energies towards O (Fig. 6a), which is favorable for reaction (2), and consequently improves the energetics for OER.9*c* For the bimetal systems of NiFeO(OH)_3_ and Ni_2_P@NiFeO(OH)_3_, the preferential acitive sites are Ni rather than Fe, as the binding energies of Fe to the OER intermediates (*OH, *O, and *OOH) are too strong to the disadvantage of the desorption (Fig. 6a). Nevertheless, the binding energy of O and OOH on the Fe-doped NiFeO(OH)_3_ can also be enhanced compared to the pure Ni(OH)_2_ and result in higher OER activity, being similar to that of the Ni_2_P@Ni(OH)_2_ heterostructure. More interestingly, after introducing both the heterostructure and Fe doping, the binding energies of OH and O on the Ni_2_P@NiFeO(OH)_3_ with Ni active sites are further optimized to get the lowest rate-limiting energy barrier of 1.722 eV (Fig. S27†), with the smallest calculated *η* value of 0.49 V compared with those of NiFeO(OH)_3_ (*η* = 0.59 V) and the hypothetical Fe-covered Ni_2_P@FeO(OH) system (*η* = 0.73 V). Interestingly, if Δ*E*_OH_–Δ*E*_O_ (the difference in binding energy between OH and O) is adopted as the descriptor, a volcano-shaped plot containing the calculated overpotential *versus* (Δ*E*_OH_–Δ*E*_O_) can be established (Fig. 6b), in which the overpotential of each model system follows the trend from our experiments, with the optimal (Δ*E*_OH_–Δ*E*_O_) value around 0.4 eV. Undoubtedly, too low and too high values of (Δ*E*_OH_–Δ*E*_O_) signify too strong binding energies of OH and O (or too weak binding energies of O and OH), being unfavorable to the formation of *O and *OH, respectively. Actually, the mixed-metal Ni_2_P@NiFeO(OH)_3_ heterostructure is located closer to the volcano summit, revealing that the heterostructure as well as the Fe-incorporation can effectively modulate the binding energies of the intermediates towards optimal values for enhancing its catalytic performance for OER.

### Electrocatalytic overall water splitting

**Fig. 7:**
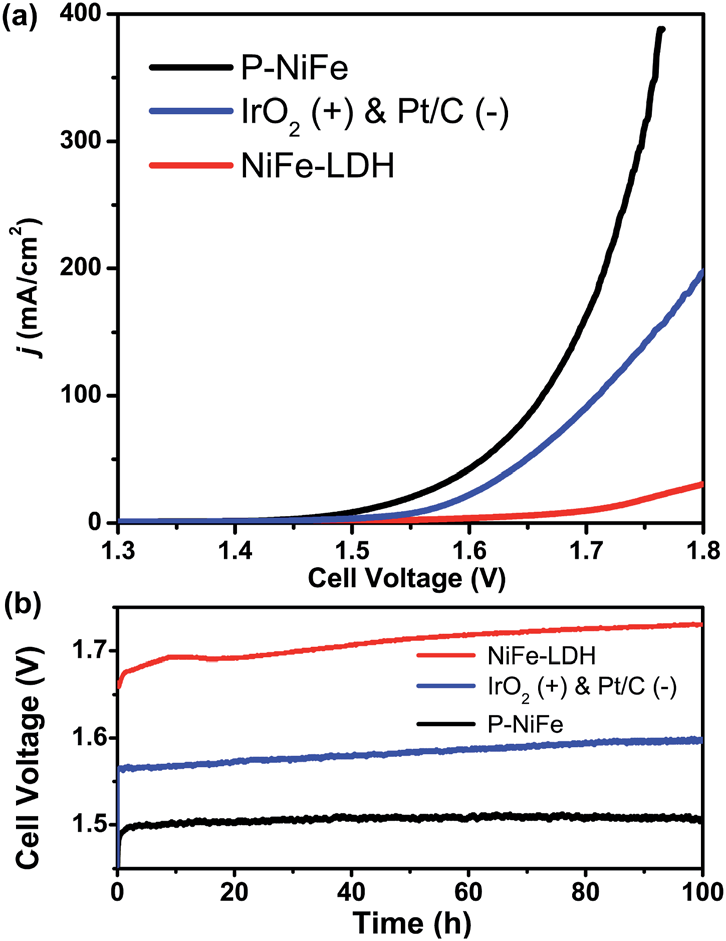
(a) LSV curves of P-NiFe (black) and NiFe-LDH (red) as HER and OER bifunctional catalysts in 1.0 M KOH solution for overall water splitting. IrO_2_ and Pt/C as OER and HER benchmarks were measured for comparison (blue). (b) Current density traces of CCE at 10 mA cm^–2^ for overall water splitting in 1.0 M KOH for P-NiFe (black), NiFe-LDH (red), and commercial benchmarks of IrO_2_ and 20% Pt/C (blue).

Based on the above results, it can be seen that P-NiFe exhibits high electrocatalytic efficiency for both HER and OER. Accordingly, two P-NiFe-based NF electrodes were utilized to construct an electrolyzer for overall water splitting. The LSV and CCE measurements indicate that it only requires a cell voltage of 1.51 V to afford a 10 mA cm^–2^ current density in 1.0 M KOH aqueous solution (Fig. 7). This performance for overall water splitting is better than those of commercial benchmarks of IrO_2_ and Pt/C on NF (1.58 V) and NiFe-LDH on NF (1.72 V). The value of 1.58 V at 10 mA cm^–2^ for an electrolyzer consisting of IrO_2_ and Pt/C is in agreement with the reported value,3b,4a,10a,10b which validates our electrochemical measurement. To our knowledge, the facilely prepared, earth-abundant P-NiFe electrocatalyst exhibits the best catalytic performance for overall water splitting among the reported non-noble electrocatalysts (including those on nickel foam substrates) in basic electrolyte (Table S2†). In addition, as shown in Fig. 7b, the P-NiFe-based electrolyzer also exhibits remarkable stability, with negligible deactivation within 100 h of CCE at 10 mA cm^–2^, and no significant change was observed in the LSV curves for overall water splitting before and after CCE (Fig. S28†). Moreover, the Faraday efficiency of the electrolyzer for overall water splitting at 10 mA cm^–2^ within 1 h was determined to be 97 ± 5% (93.1 ± 4.5 μmol) for H_2_ and 99 ± 5% (46.6 ± 2.3 μmol) for O_2_, respectively, confirming the high electrocatalytic stability and efficiency of P-NiFe nanosheets. Furthermore, the above as-established electrolyzer could continuously release H_2_ and O_2_ bubbles with a single-cell AAA battery of 1.5 V (see video in the ESI†).

## Conclusion

Our studies have revealed that the nickel element in NiFe-LDH can be selectively phosphorized and extracted into Ni_2_P nanoparticles to generate an Ni_2_P@FePO_*x*_ heterostructure, which is further transformed into a new heterostructure of P-NiFe comprised of NiFe hydroxide around the inner Ni_2_P nanoparticles through a self-optimization of the Ni_2_P@FePO_*x*_ during the electrocatalytic process. The *in situ* formed P-NiFe electrocatalyst exhibits high electrocatalytic activity and stability for both HER and OER in 1.0 M KOH, and can be used as a bifunctional electrocatalyst for overall water-splitting, with a cell voltage of only 1.51 V at a current density of 10 mA cm^–2^. Its electrocatalytic activity is among the most active earth-abundant bifunctional electrocatalysts reported to date, and is even superior to the state-of-the-art noble-metal coupled electrocatalysts of IrO_2_ and Pt/C on NF (1.58 V at 10 mA cm^–2^). The results of theoretical calculations suggest that the *in situ* formed P-NiFe heterostructure could optimize the adsorption energies for both HER and OER intermediates, thus dramatically enhancing its electrocatalytic activity. This facile and efficient strategy will open up new avenues for the construction of high-performance and low-cost electrocatalysts for overall water splitting to store intermittent solar energy and wind energy sources into hydrogen fuel.

## Conflicts of interest

There are no conflicts to declare.

## Supplementary Material

Supplementary information

Supplementary movie
