# Supplementary material for: Extraction of nickel from NiFe-LDH into Ni2P@NiFe hydroxide as a bifunctional electrocatalyst for efficient overall water splitting
Source: Chem Sci. 2017 Dec 21;9(5):1375–84. doi: 10.1039/c7sc04569g (PMC5885978; doi:10.1039/c7sc04569g)
Supplement: Supplementary file 1 [file SC-009-C7SC04569G-s001.pdf]

Electronic Supplementary Material (ESI) for Chemical Science.
This journal is © The Royal Society of Chemistry 2017

Supporting Information

Extraction of nickel from NiFe-LDH into Ni_2P@NiFe hydroxide as a bifunctional electrocatalyst for efficient overall water splitting

Fang-Shuai Zhang,‡[a] Jia-Wei Wang,‡[b] Jun Luo,[b] Rui-Rui Liu,[b] Zhi-Ming Zhang,*[b] Chun-Ting He,*[a] and Tong-Bu Lu*[ab]

Table S1. A comparison of the OER overpotentials for the reported electrocatalysts.[a]

Catalysts	η at 10 mAcm^{-2} (mV)[c]	η at 100 mAcm^{-2} (mV)	Tafel slope (mVdec^{-1})	Mass loading (mg cm^{-2})	Substrate[b]	References
P-NiFe	205	230	32	~1.0	NF	This work
NiFe-LDH	250	280	70	~1.0	NF	This work
NiFe-LDH@r-GO	225	N.A.	39	0.25	NF	*Angew. Chem. Int. Ed.* **2014**, *53*, 7584.
NiFe-LDH@CNT	~260	N.A.	31	0.25	CFP	*J. Am. Chem. Soc.* **2013**, *135*, 8452.
$Fe(PO_3)_2$	177[d]	221	51.9	~8.0	NF	*PNAS***2017**, *114*, 5607.
Gelled FeCoW	191	~250	37	0.21	NF	*Science* **2016**,*352*, 333.
CoP@ rGO	280	440	75	0.28	GCE	*J. Am. Chem. Soc.* **2016**, *138*, 14686.
CoMnP	330	N.A.	61	0.28	GCE	*J. Am. Chem. Soc.* **2016**, *138*, 4006.
NiCoP@C	330	N.A.	96	0.25	GCE	*Angew. Chem. Int. Ed.* **2017**, *56*, 3897.
NiFeSe	N.A.	270	47.2	~1.5	NF	*ACS Appl. Mater. Interfaces* **2016**, 8, 19386
Pulse-Electrodeposited NiFeOOH	260	N.A.	N.A.	~0.1	Au	*ACS Catal.* **2015**, *5*, 6680.
Electro-oxidized $Co_{0.37}Ni_{0.26}Fe_{0.37}O$	232	~280	38	N.A.	CC	*ACS Cent. Sci.* **2015**, *1*, 244.
$NiFeO_x$	230	260	31.5	1.5	CFP	*Nat. Commun. **2015**, 6, 7261.*
Fe-doped Ni_3S_2	N.A.	253	65.5	~7.9	NF	*J. Mater. Chem. A* **2015**, *3*, 23207.
Co_3O_4@C nanowire array	220	N.A.	61	~0.2	Cu foil	*J. Am. Chem. Soc.* **2014**, *136*, 13925.
MoS_2/Ni_3S_2	218	290	88	9.7	NF	*Angew. Chem. Int. Ed.* **2016**, *55*, 6702.
CoMn-LDH	324	N.A.	43	0.142	GCE	*J. Am. Chem. Soc.* **2014**, *136*, 16481.
$CoFe_2O_4$/C NRAs	240	290	45	1.03	NF	*Adv. Mater.* **2017,** *29*, 1604437.
Co_4N nanowire array	257	N.A.	44	~0.82	CC	*Angew. Chem. Int. Ed.* **2015**, *54*, 14710.
FeP@rGO	260	N.A.	175	~0.71	CFP	*J. Mater. Chem. A* **2016**, *4*, 9750.
Ni_3Se_2[c]	290	N.A.	142	0.022	NF	*Energy Environ. Sci.* **2016**, *9*, 1771.
Ni_3C@C	~320	N.A.	46	0.285	GCE	*Adv. Mater.* **2016**, *28*, 3326.
$W_{0.5}Co_{0.4}Fe_{0.1}$	250	310	32	N.A.	NF	*Angew. Chem. Int. Ed.***2017**, *56*, 4502.
$CoTe_2$	357	N.A.	32	0.25	GCE	*Angew. Chem. Int. Ed.***2017**, 10.1002/anie.201701531.
CoAl-LDH	252	N.A	36	0.05	GCE	*Adv. Mater.***2016**, *28*, 7640.

a) The electrolyte is 1.0 M KOH unless otherwise stated. η is overpotential.
b) NF = nickel foam; GCE = glassy carbon electrode; CFP = carbon fiber paper; CC = carbon cloth.
c) The overpotentials obtained from the chronoamperometric measurements.
d) The overpotentials obtained from Tafel slope.

Table S2. A comparison of the reported bifunctional electrocatalysts on nickel foam (NF)for overall water splitting in 1.0 M KOH (or NaOH) solution.

Catalysts	OER η@10 mAcm^{-2} (mV)	HER η@10 mAcm^{-2} (mV)	Cell voltage (10 mAcm^{-2} for overall water splitting) (V)	Substrate	Reference
P-NiFe	205	75	1.51	NF	This work
NiFe-LDH	250	230	1.73	NF	
IrO_2 (+) / Pt/C (-)	320	45	1.58	NF	
NiFe-LDH	240	210	1.70	NF	*Science* **2014**, *345*, 1593.
NiFe-LDH@ DG10	210	115@20 mAcm^{-2}	1.5@20 mAcm^{-2}	NF	*Adv. Mater.* **2017**, *29*, 1700017.
Porous MoO_2	260	25	1.53	NF	*Adv. Mater.* **2016**, *28*, 3785.
NiSe nanowire	N.A.	96	1.63	NF	*Angew. Chem. Int. Ed.* **2015**, *54*, 9351.
Ni_2P	290	220	1.63 (5 mg cm^{-2} loading)	NF	*Energy Environ. Sci.* **2015**, *8*, 2347.
$Ni_{2/3}Fe_{1/3}$@rGO	240	~550	N.A.	NF	*ACS Nano* **2015**, *9*, 1977.
Ni_3FeN nanoparticles	280	158	N.A.	NF	*Adv. Energy Mater.* **2016**, *6*, 1502585.
Fe- and O-doped Co_2P	274	88	1.54	NF	*ACS Nano* **2016**, 10, 8738.
NiCoP	280	35	1.58	NF	*Nano Lett.* **2016**, *16*, 7718
MoS_2/Ni_3S_2	218	110	1.56	NF	*Angew. Chem. Int. Ed.* **2016**, *55*, 6702.
Co-Mn carbonate hydroxide	N.A.	180	1.68	NF	*J. Am. Chem. Soc.* **2017**, *139*, 8320.

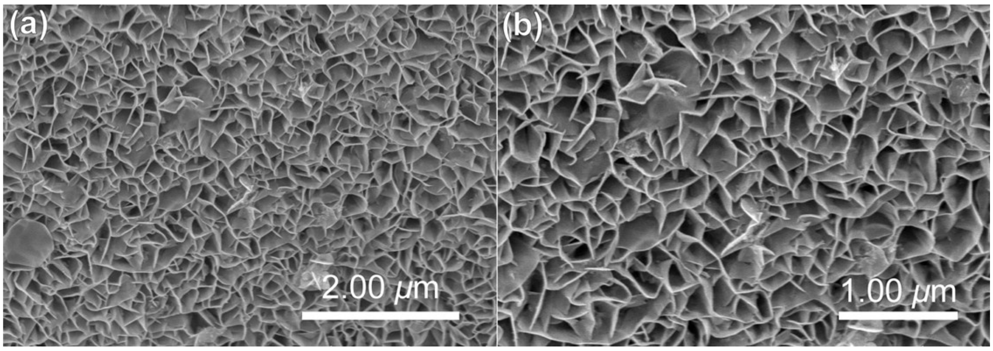

Fig. S1. SEM images of NiFe-LDH nanosheets on NF.

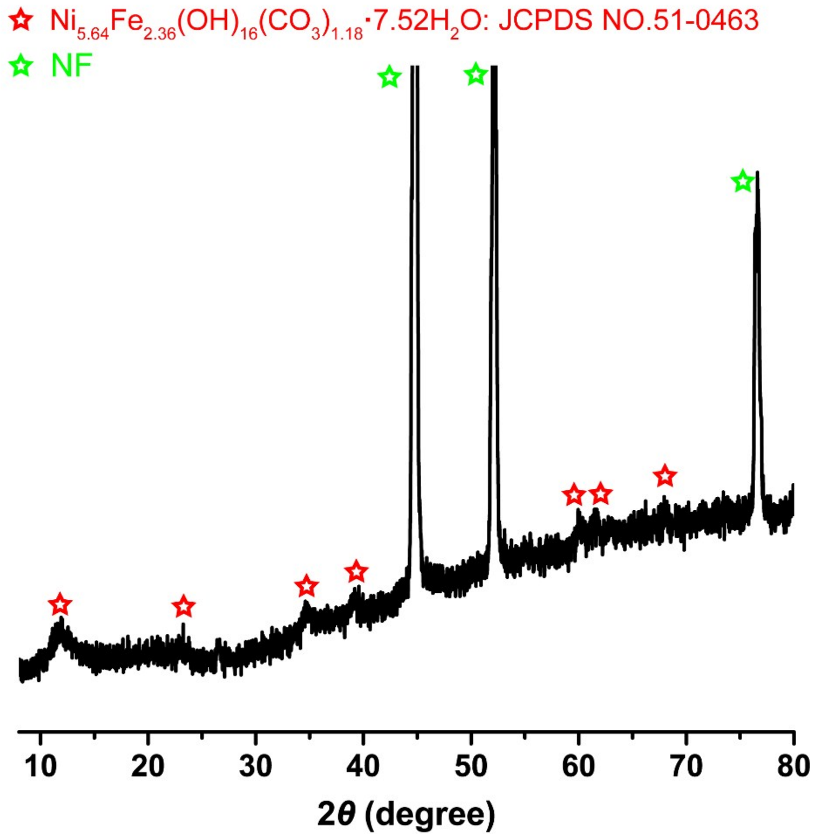

Fig. S2. XRD pattern for NiFe-LDH.

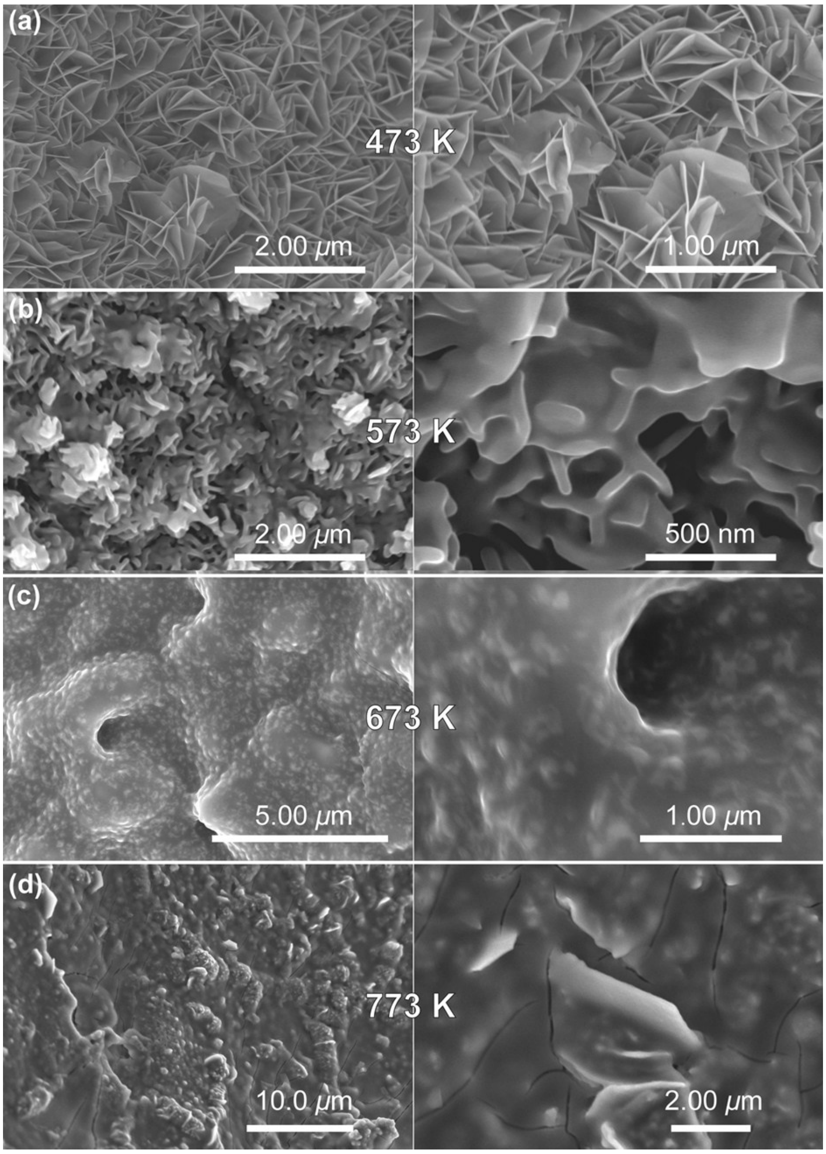

Fig. S3. SEM images of $Ni_2P@FePO_x$ samples prepared at the phosphorization temperature of a) 473, b) 573, c) 673 and d) 773 K for 3 h.

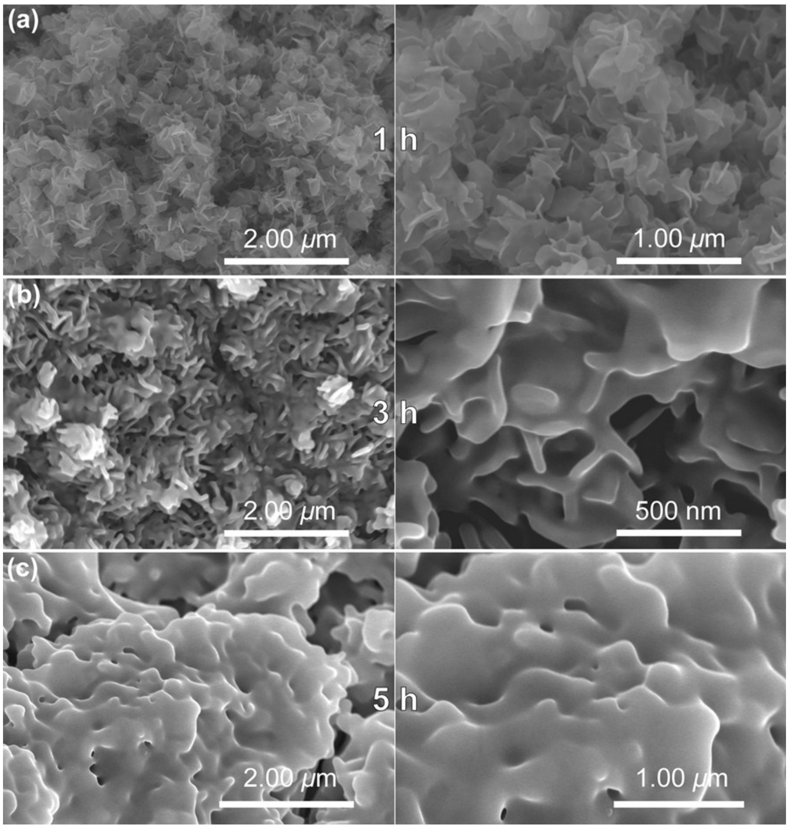

Fig. S4. SEM images of $Ni_2P@FePO_x$ samples prepared with varying time scales (1, 3 and 5 h) of phosphorization at 573 K. a) 1, b) 3 and c) 5 h.

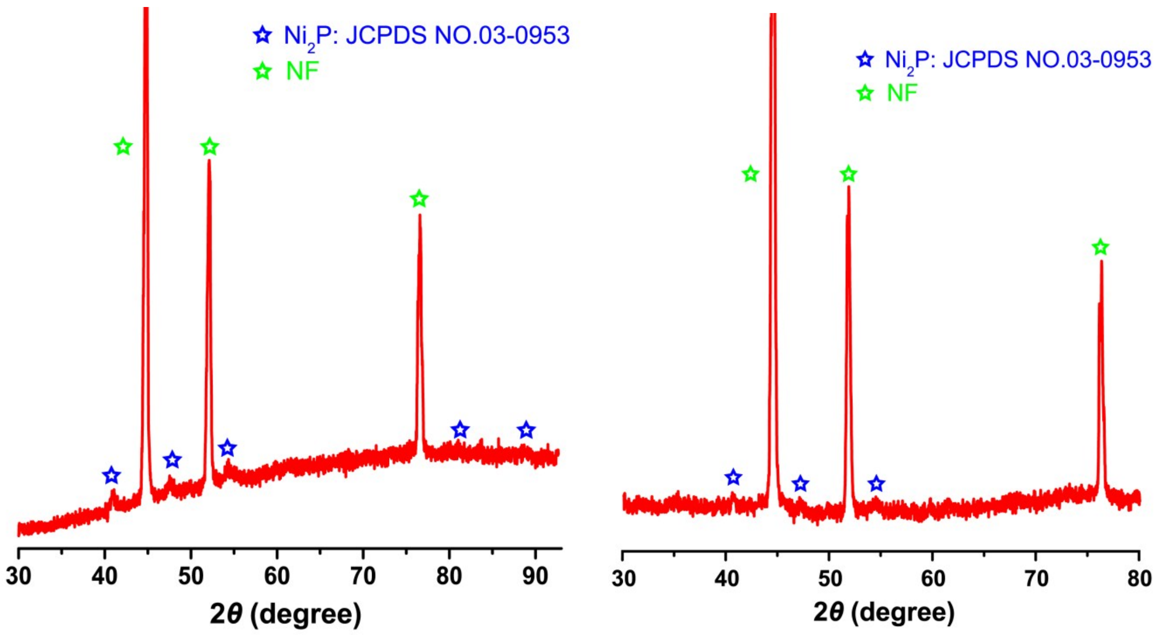

Fig. S5. XRD patterns for $Ni_2P@FePO_x$ (left) and P-Ni (right), demonstrating the existence of Ni_2P species in $Ni_2P@FePO_x$ and P-Ni.

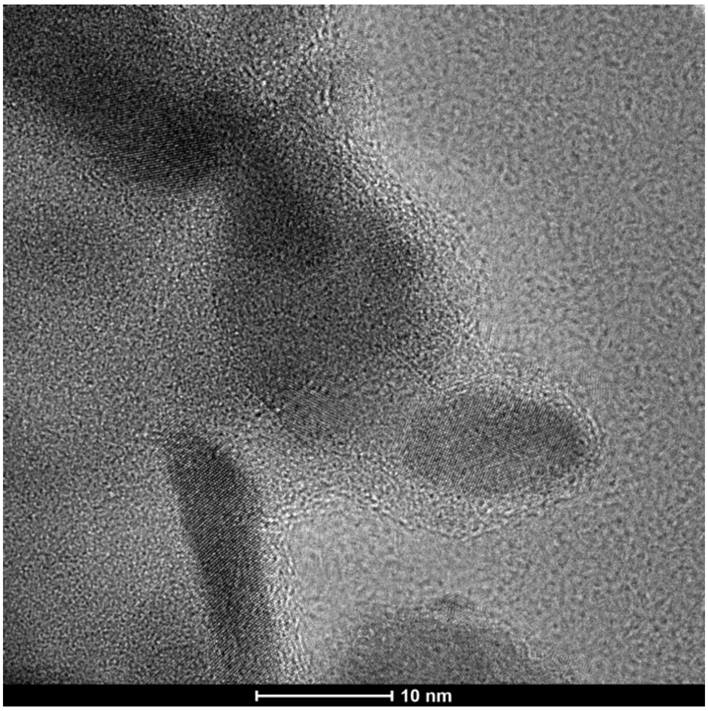

Fig. S6. HRTEM image of $Ni_2P@FePO_x$ sample, showing the crystalline nanoparticles are embedded in the amorphous substrate.

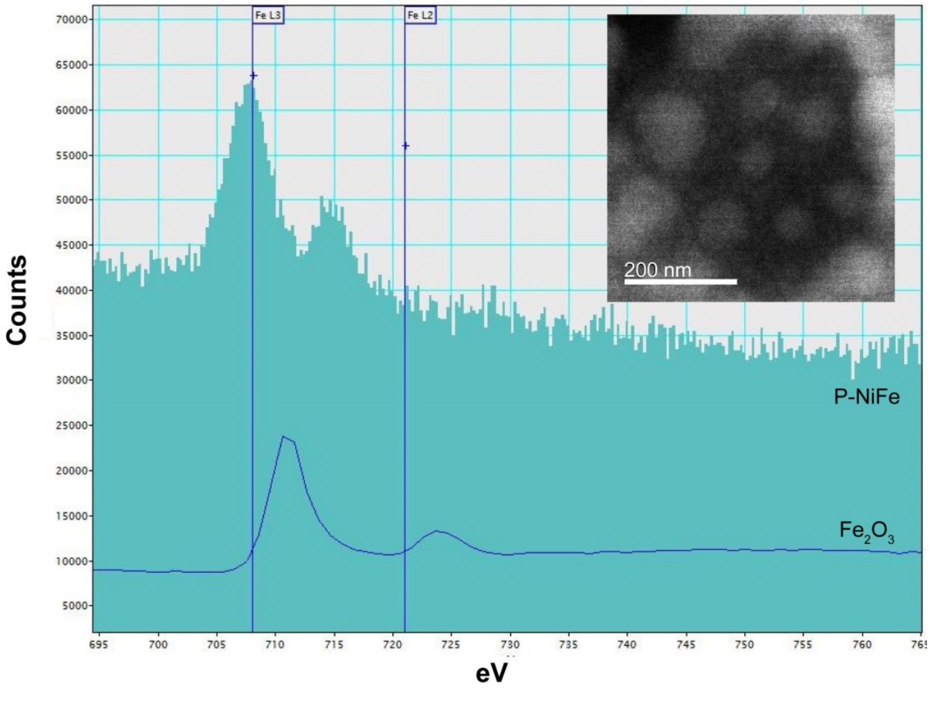

Fig. S7. EELS of iron in $Ni_2P@FePO_x$ with the comparison of Fe_2O_3 sample, indicating that the amorphous substrate is not the Fe_2O_3 species.

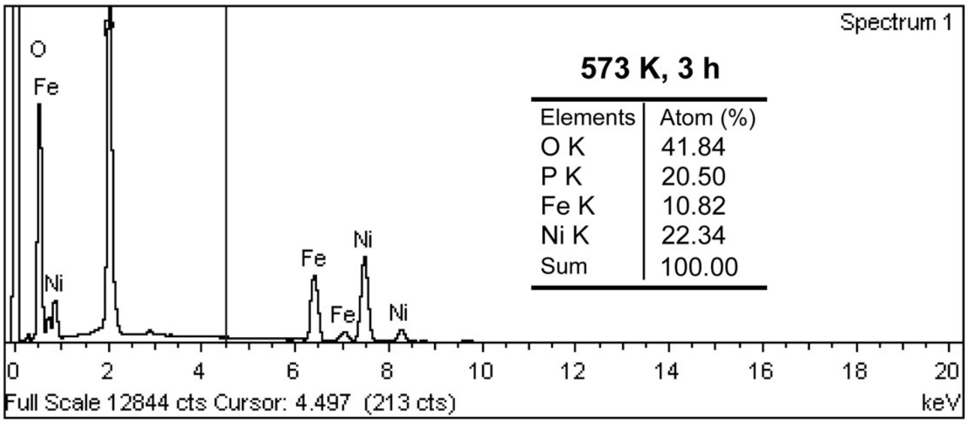

Fig. S8. EDS of $Ni_2P@FePO_x$ sample by SEM.

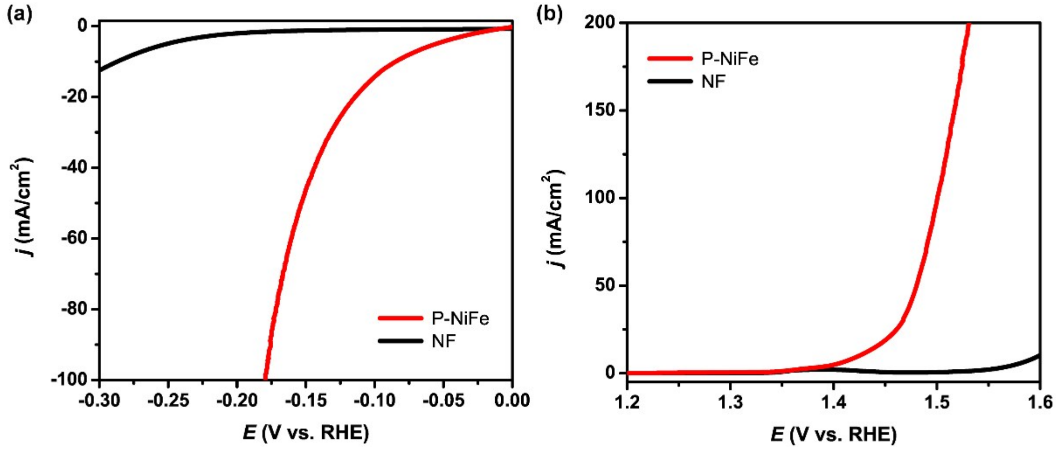

Fig. S9. LSV curves for P-NiFe (red) and bare NF (black) at the scan rate of 5 mV/s for a) HER, and b) OER.

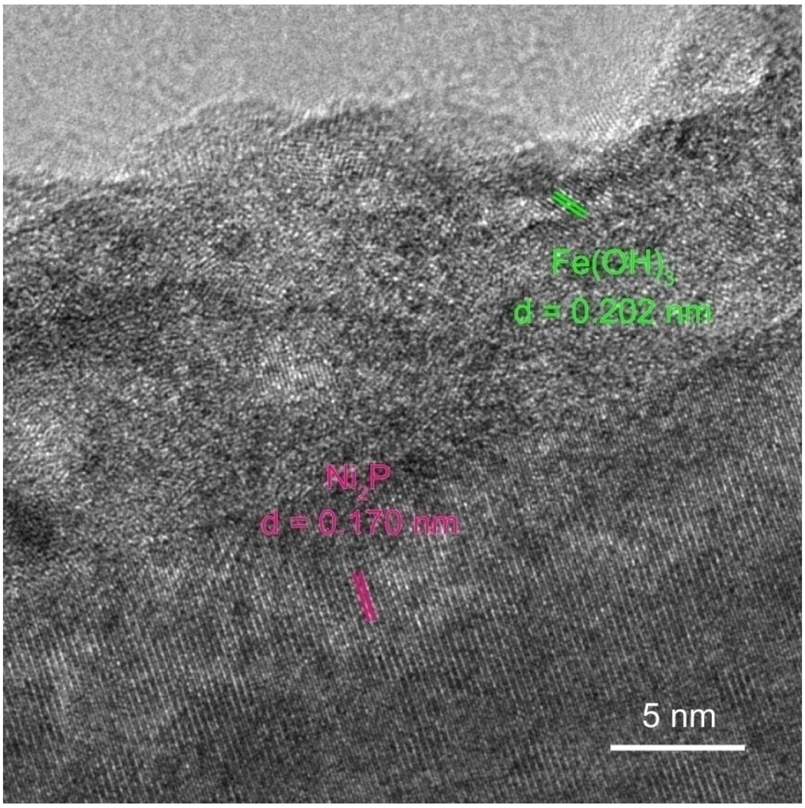

Fig. S10. HRTEM image of P-NiFe after 25 h of CCE for HER.

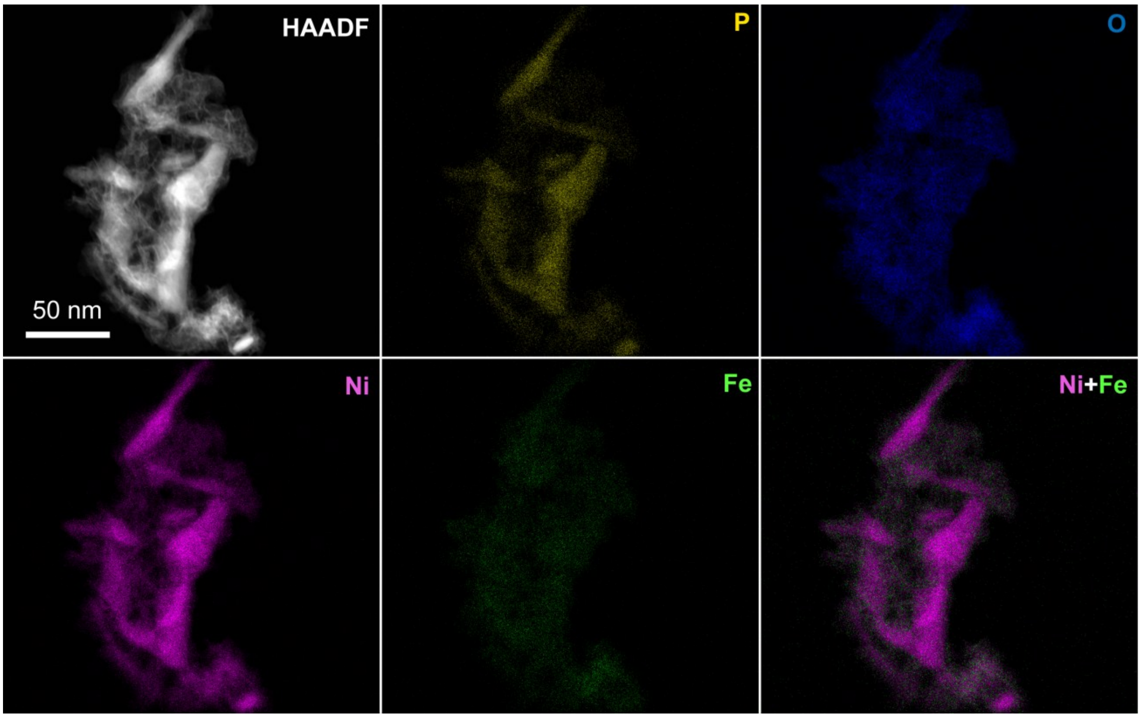

Fig. S11. HAADF-STEM image and EDS elemental mapping images for P-NiFe after 25 h of CCE for HER.

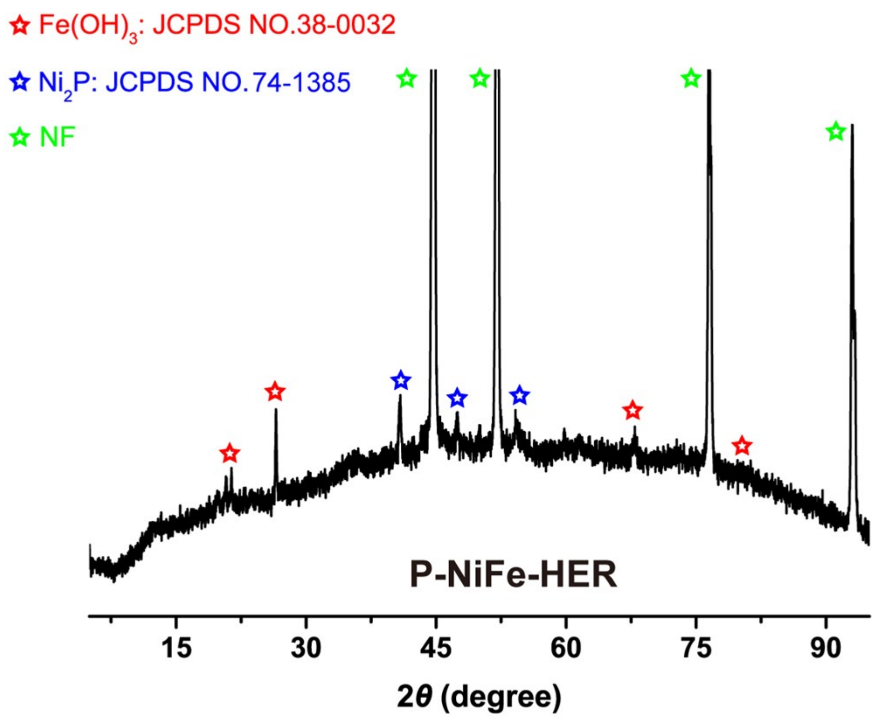

Fig. S12. XRD pattern of P-NiFe after 25 h of CCE for HER.

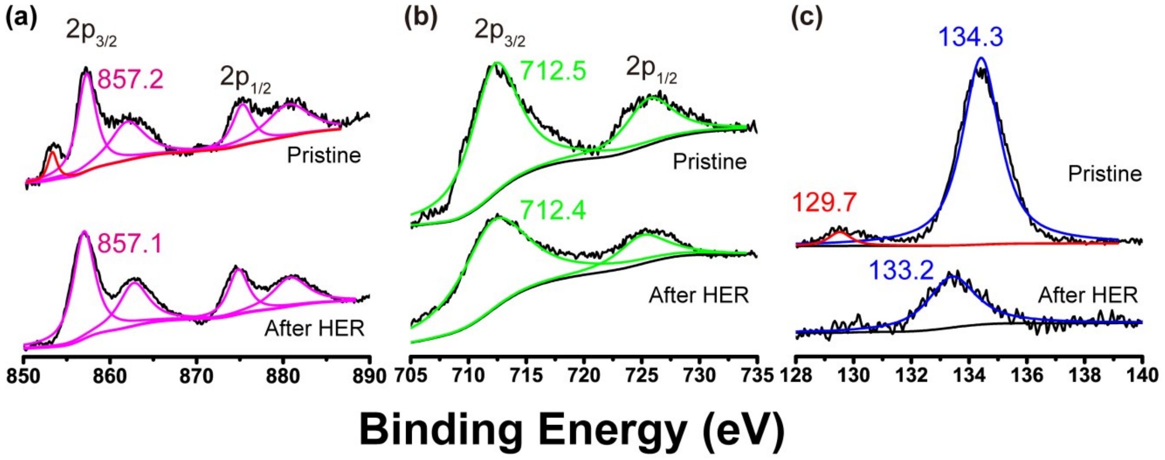

Fig. S13. XPS results for as-prepared $Ni_2P@FePO_x$ (top) and P-NiFe after 25 h of CCE for HER (bottom), showing a) Ni 2p, b) Fe 2p and c) P 2p signals.

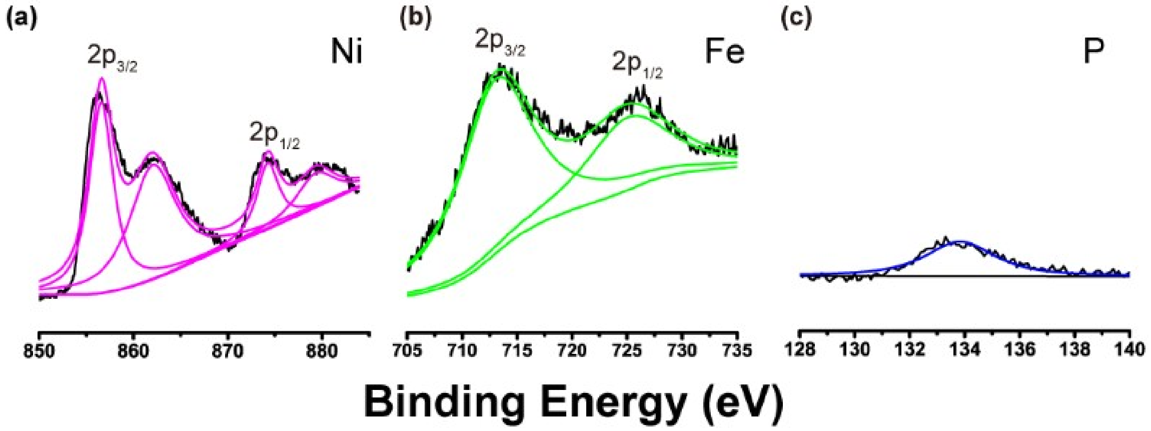

Fig. S14. XPS results for P-NiFe after LSV measurement and before CCE for HER, indicating the surface oxidation of Ni_2P into $Ni(OH)_2$ occurs rapidly in solution.

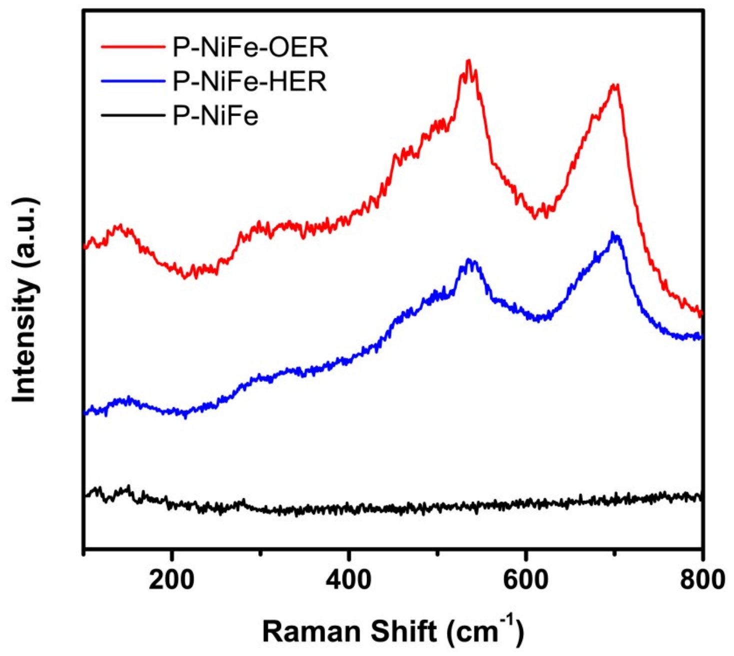

Fig. S15. Raman spectra for P-NiFe, and P-NiFe after 25 h of CCE for HER and OER, respectively.

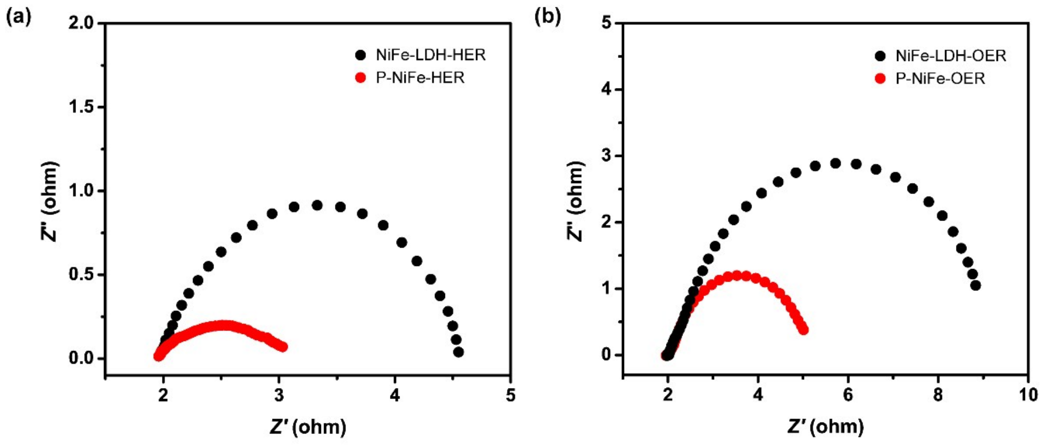

Fig. S16. EIS plots of P-NiFe (red) and NiFe-LDH (black) after 25 h of CCE for HER a) and OER b), respectively.

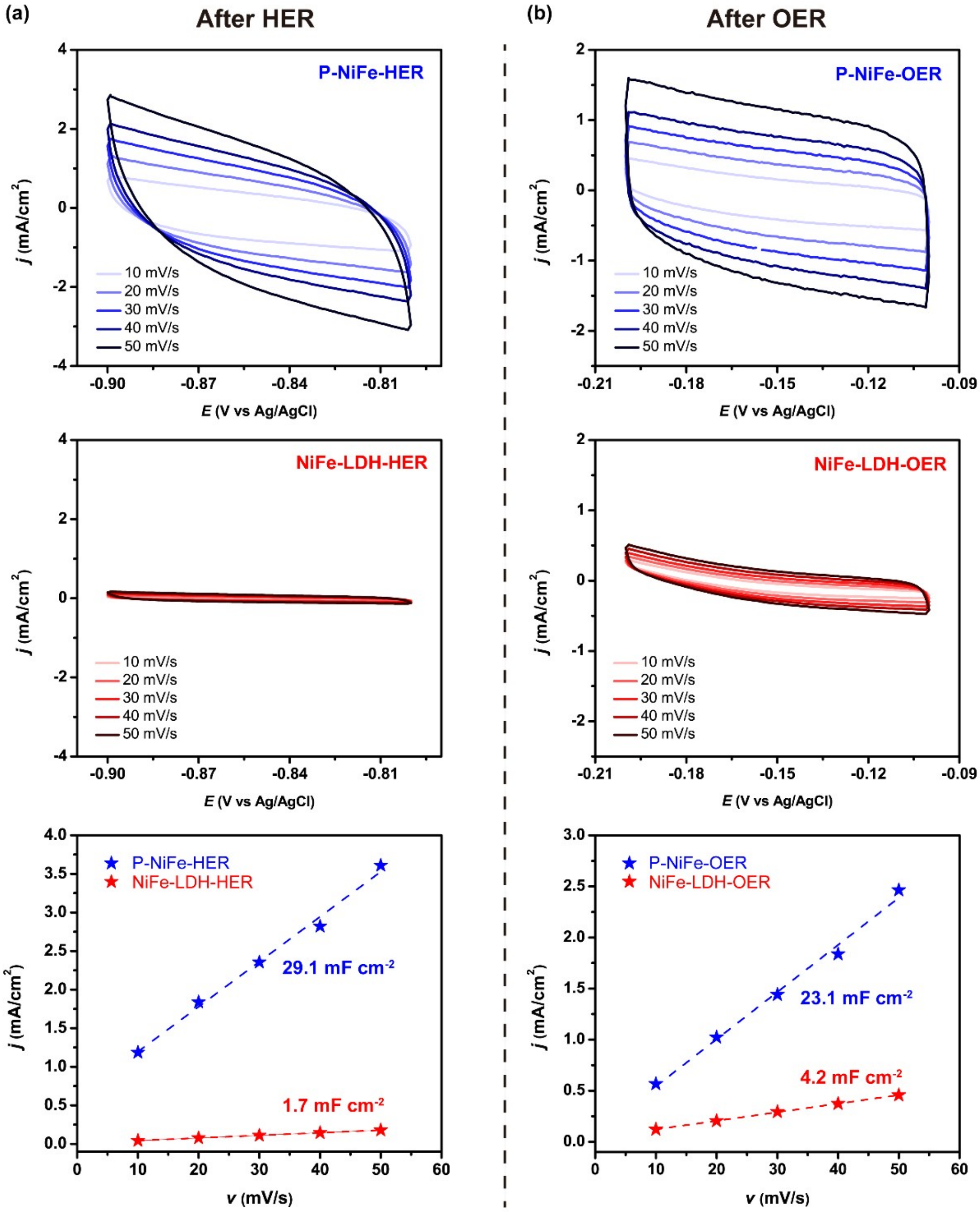

Fig. S17. CVs of P-NiFe (blue) and NiFe-LDH (red) after 25 h of CCE for HER a) and OER b) in 1.0 M KOH solution at varying scan rates, with the plots of the capacitive current density (j) as a function of scan rate (v).

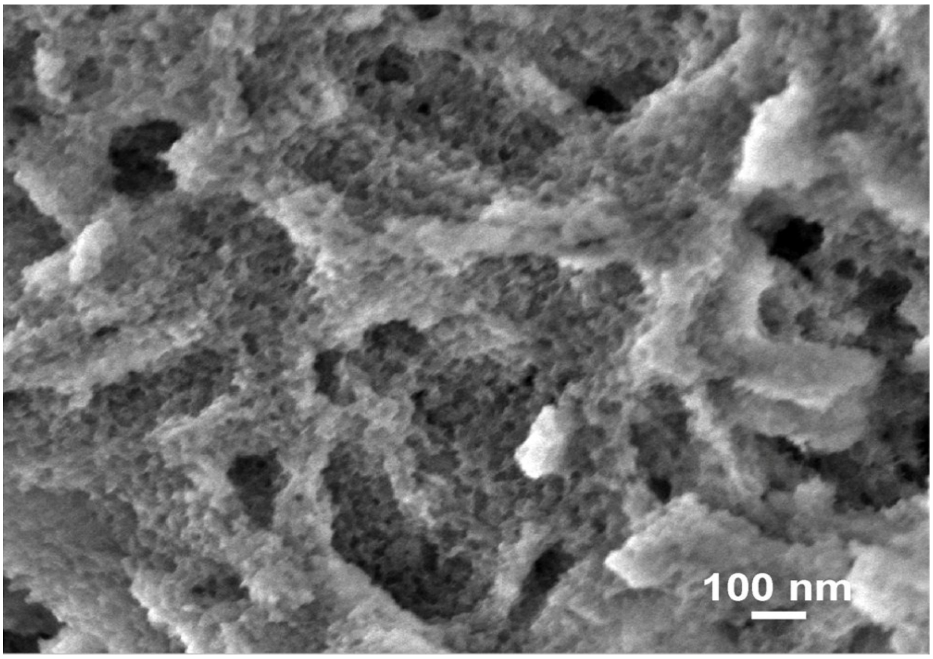

Fig. S18. SEM image of P-NiFe after 25 h of CCE for HER in 1.0 M KOH.

Ni_2P		
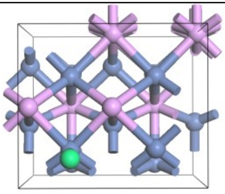	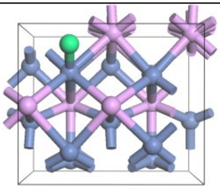	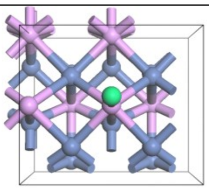
ΔG_{H*} = 0.327 eV on planar Ni site	ΔG_{H*} = 0.325 eV on square pyramidal Ni site	ΔG_{H*} = 0.335 eV on P site
$Ni(OH)_2$		
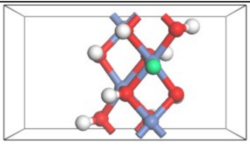	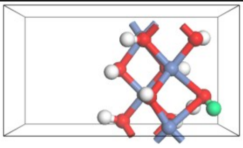	
ΔG_{H*} = 0.635 eV on Ni site	ΔG_{H*} = 1.049 eV on O site	
FeO(OH)		
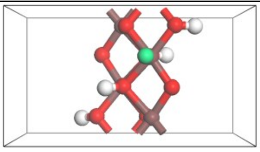	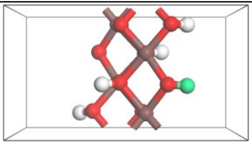	
ΔG_{H*} = 0.614 eV on Fe site	ΔG_{H*} = -0.706 eV on O site	

$NiFeO(OH)_3$ (NiFe-LDH)		
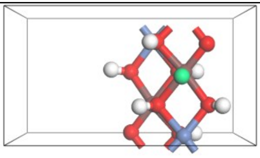	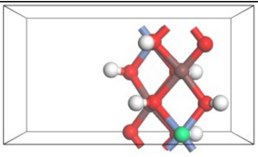	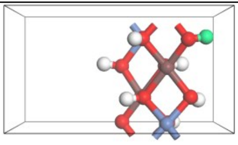
ΔG_{H*} = 0.346 eV on Fe site	ΔG_{H*} = 0.485 eV on Ni site	ΔG_{H*} = -0.659 eV on O site
$Ni_2P@Ni(OH)_2$		
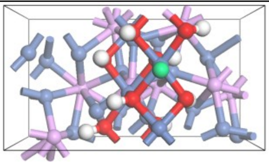		
ΔG_{H*} = -0.198 eV on Ni site		
$Ni_2P@FeO(OH)$		
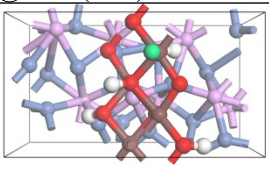	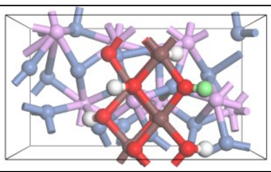	
ΔG_{H*} = 0.754 eV on Fe site	ΔG_{H*} = -0.355 eV on O site	
$Ni_2P@NiFeO(OH)_3$ (P-NiFe after HER)		
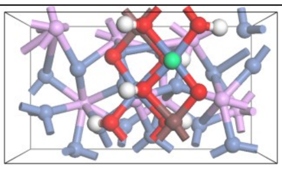	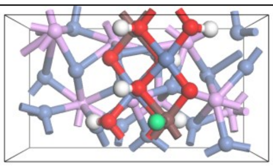	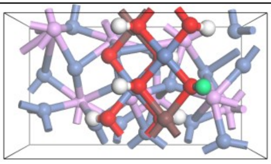
ΔG_{H*} = 0.063 eV on Ni site	ΔG_{H*} = 0.143 eV on Fe site	ΔG_{H*} = -0.338 eV on O site

Fig. S19. Calculated free energies of H* adsorption on the surface of different catalytic active sites of the catalysts.

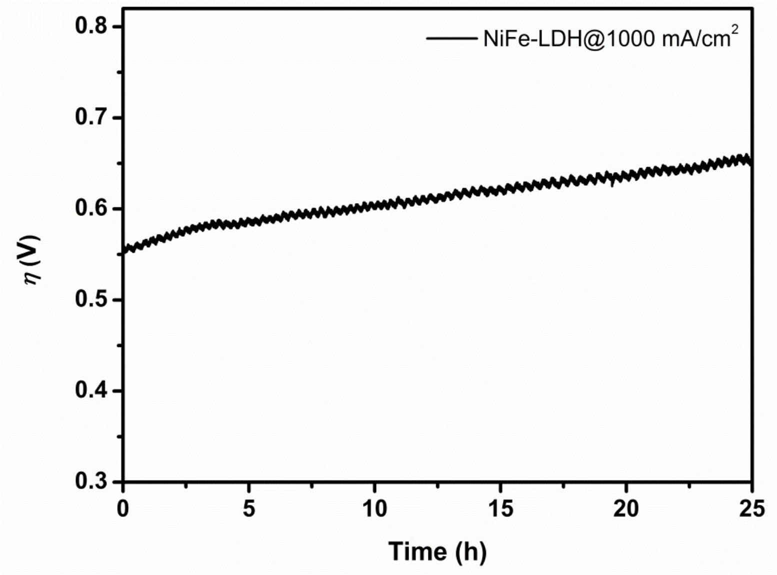

Fig. S20. Current density trace of CCE at 1000 mA/cm^2 for NiFe-LDH in 1.0 M KOH.

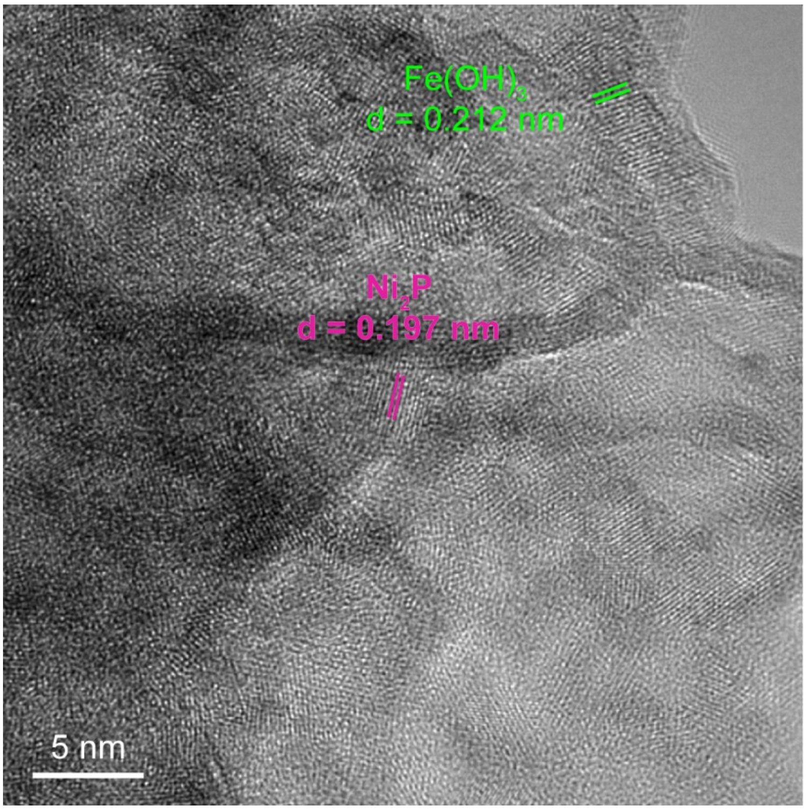

Fig. S21. HRTEM image of P-NiFe after 25 h of CCE for OER.

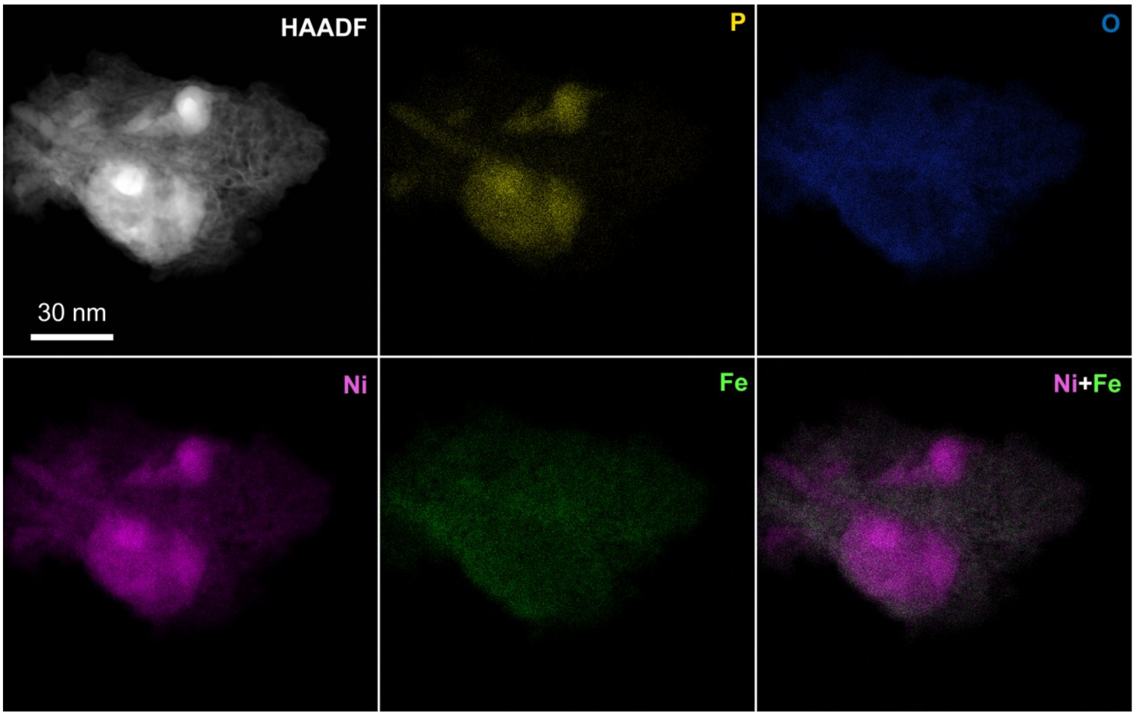

Fig. S22. EDS elemental mapping images of P-NiFe after 25 h of CCE for OER by STEM-HAADF.

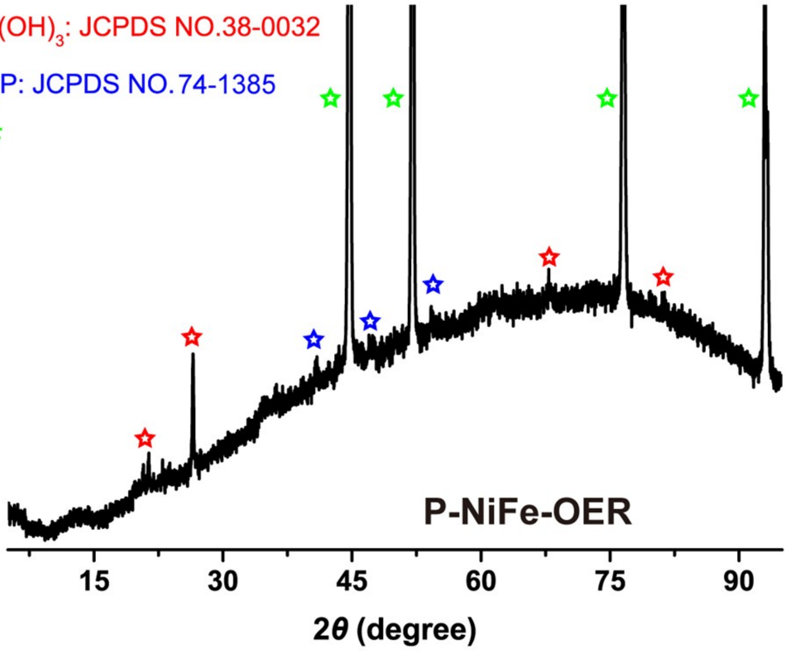

Fig. S23. XRD pattern of P-NiFe after 25 h of CCE for OER.

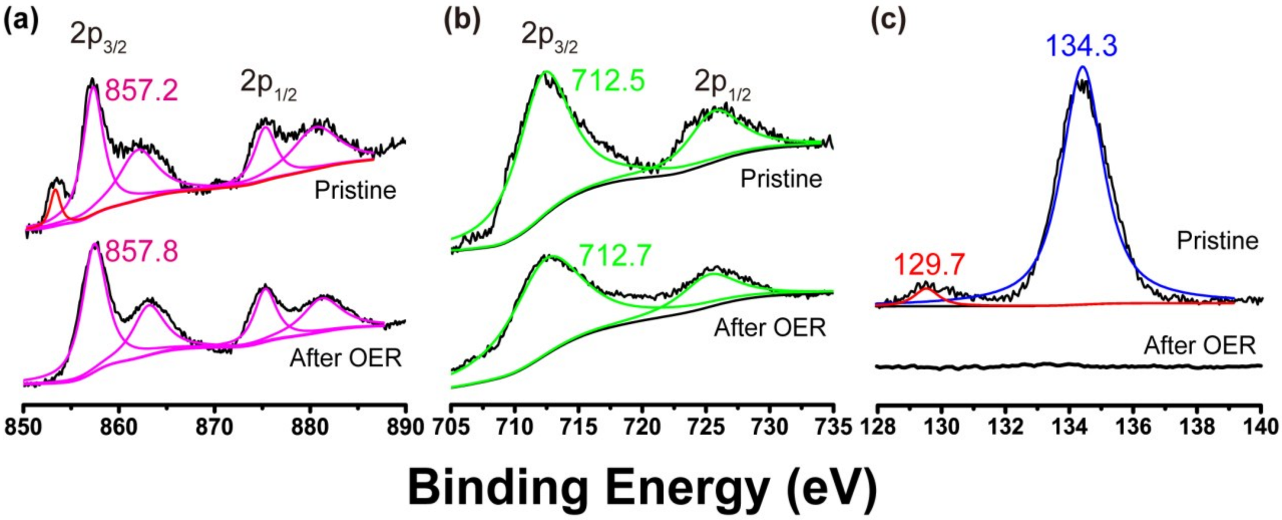

Fig. S24. XPS results for as-prepared P-NiFe (top) and P-NiFe after 25 h of CCE for OER (bottom), showing a) Ni 2p, b) Fe 2p and c) P 2p signals.

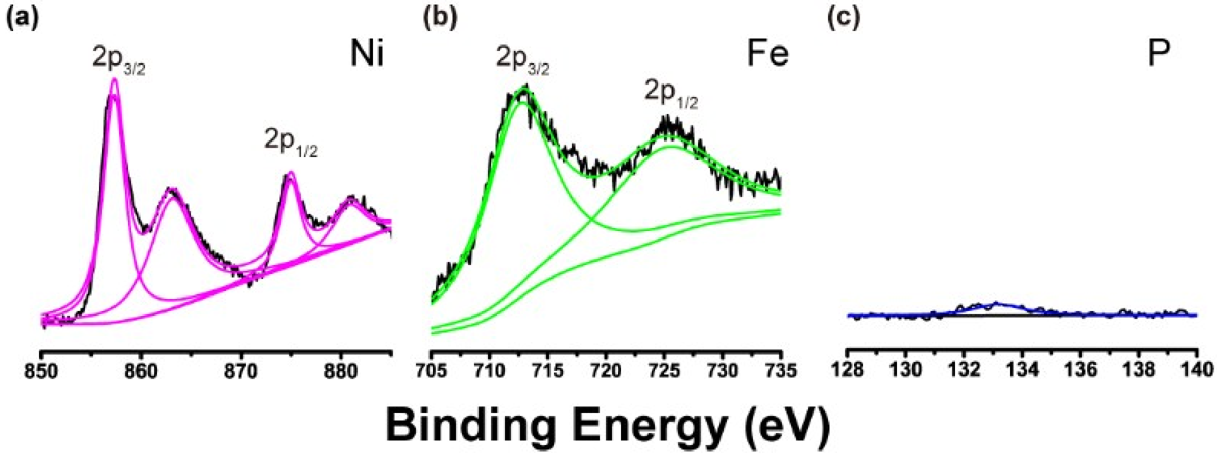

Fig. S25. XPS results for P-NiFe after LSV measurement and before CCE for OER, indicating the surface oxidation of Ni_2P into $Ni(OH)_2$ occurs rapidly in solution.

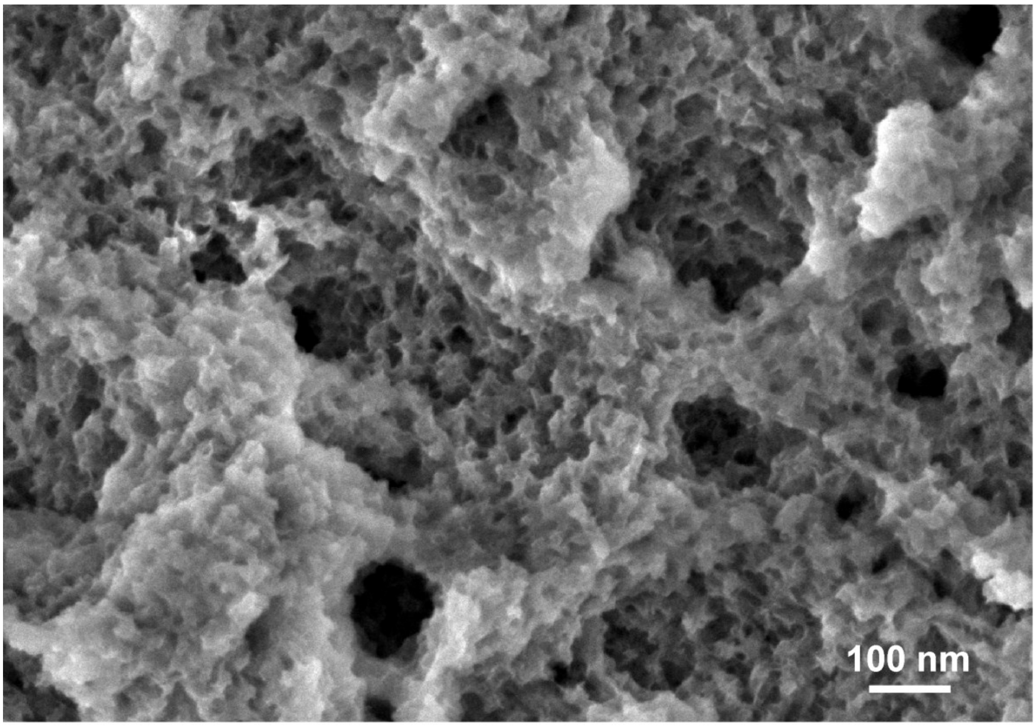

Fig. S26. SEM image of P-NiFe after 25 h of CCE for OER in 1.0 M KOH.

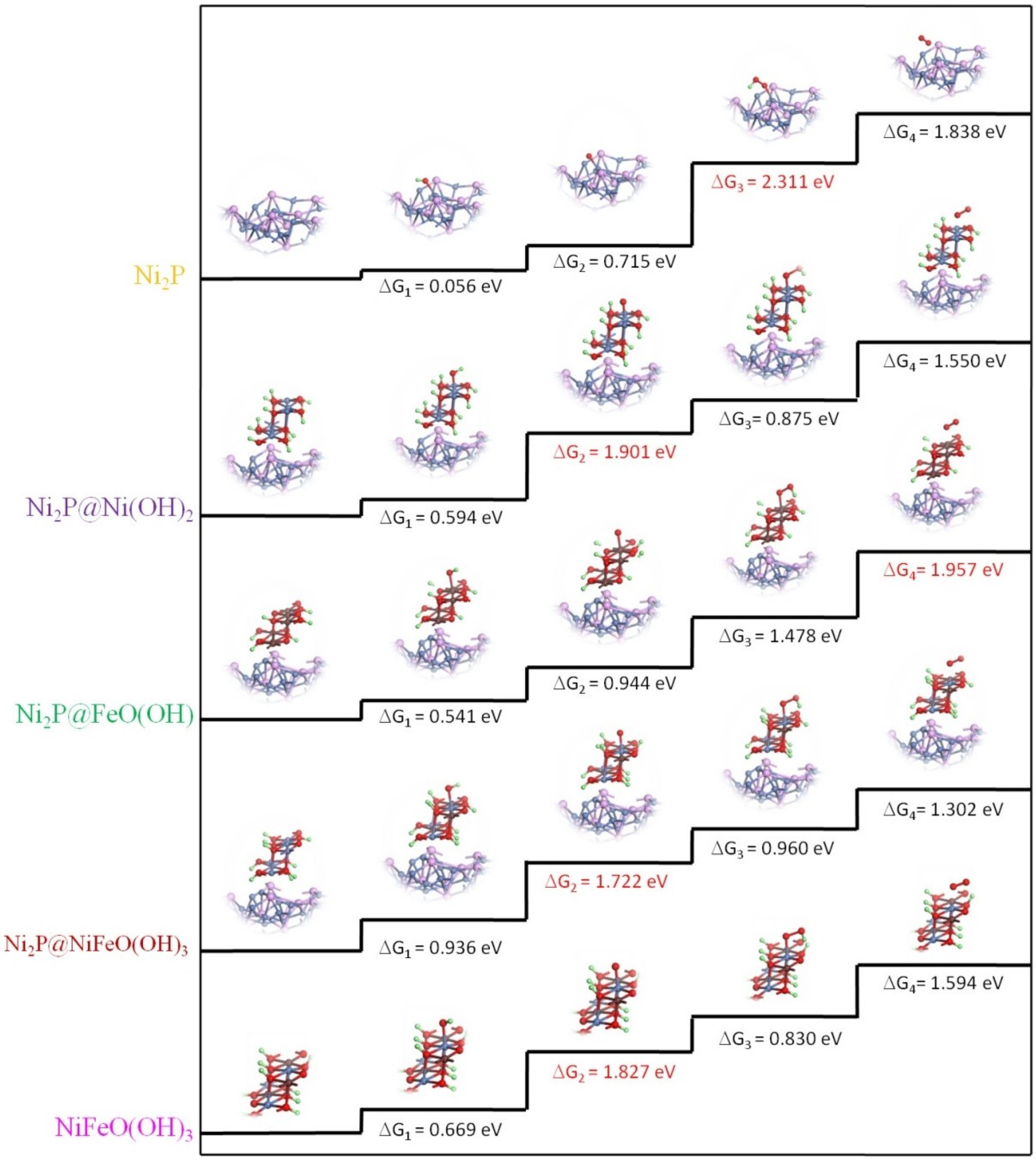

Fig. S27. DFT calculation. The primitive steps and energy diagram of the OER process on the model surface structure of Ni_2P, $Ni_2P@Ni(OH)_2$, $Ni_2P@FeO(OH)_3$, $NiFeO(OH)_3$ (NiFe-LDH), and $Ni_2P@NiFeO(OH)_3$ (P-NiFe after OER).

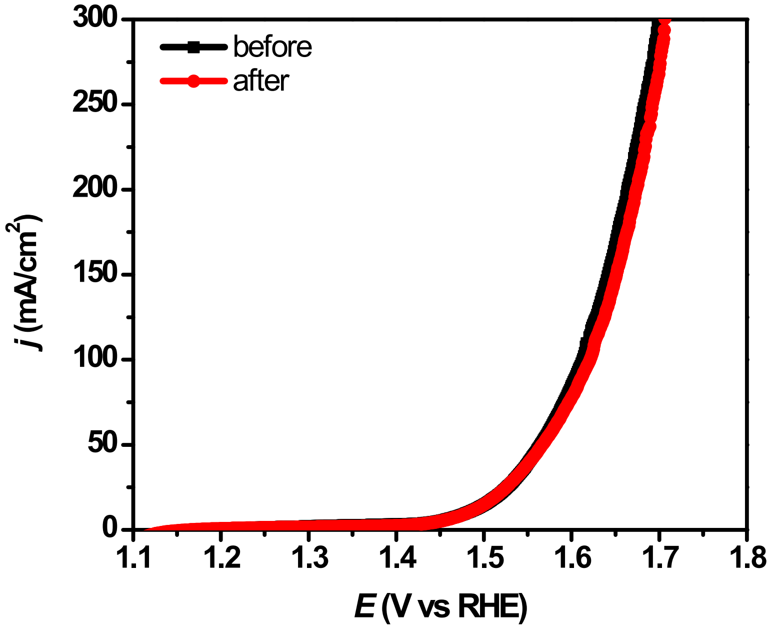

Fig. S28. LSV curves for P-NiFe before (black) and after (red) 100 h of overall water splitting at 10 mA/cm^2.
